# Comparative Analysis of the Nodule Transcriptomes of *Ceanothus thyrsiflorus* (Rhamnaceae, Rosales) and *Datisca glomerata* (Datiscaceae, Cucurbitales)

**DOI:** 10.3389/fpls.2018.01629

**Published:** 2018-11-14

**Authors:** Marco G. Salgado, Robin van Velzen, Thanh Van Nguyen, Kai Battenberg, Alison M. Berry, Daniel Lundin, Katharina Pawlowski

**Affiliations:** ^1^Department of Ecology, Environment and Plant Sciences, Stockholm University, Stockholm, Sweden; ^2^Laboratory of Molecular Biology, Department of Plant Sciences, Wageningen University, Wageningen, Netherlands; ^3^Department of Plant Sciences, University of California, Davis, Davis, CA, United States; ^4^Centre for Ecology and Evolution in Microbial Model Systems, Linnaeus University, Kalmar, Sweden; ^5^Department of Biochemistry and Biophysics, Stockholm University, Stockholm, Sweden

**Keywords:** nitrogen-fixing root nodules, actinorhiza, nitrogen metabolism, divergent evolution, subtilase, defensin, Nod factor receptor

## Abstract

Two types of nitrogen-fixing root nodule symbioses are known, rhizobial and actinorhizal symbioses. The latter involve plants of three orders, Fagales, Rosales, and Cucurbitales. To understand the diversity of plant symbiotic adaptation, we compared the nodule transcriptomes of *Datisca glomerata* (Datiscaceae, Cucurbitales) and *Ceanothus thyrsiflorus* (Rhamnaceae, Rosales); both species are nodulated by members of the uncultured *Frankia* clade, cluster II. The analysis focused on various features. In both species, the expression of orthologs of legume Nod factor receptor genes was elevated in nodules compared to roots. Since arginine has been postulated as export form of fixed nitrogen from symbiotic *Frankia* in nodules of *D. glomerata*, the question was whether the nitrogen metabolism was similar in nodules of *C. thyrsiflorus*. Analysis of the expression levels of key genes encoding enzymes involved in arginine metabolism revealed up-regulation of arginine catabolism, but no up-regulation of arginine biosynthesis, in nodules compared to roots of *D. glomerata*, while arginine degradation was not upregulated in nodules of *C. thyrsiflorus*. This new information corroborated an arginine-based metabolic exchange between host and microsymbiont for *D. glomerata*, but not for *C. thyrsiflorus.* Oxygen protection systems for nitrogenase differ dramatically between both species. Analysis of the antioxidant system suggested that the system in the nodules of *D. glomerata* leads to greater oxidative stress than the one in the nodules of *C. thyrsiflorus*, while no differences were found for the defense against nitrosative stress. However, induction of nitrite reductase in nodules of *C. thyrsiflorus* indicated that here, nitrite produced from nitric oxide had to be detoxified. Additional shared features were identified: genes encoding enzymes involved in thiamine biosynthesis were found to be upregulated in the nodules of both species. Orthologous nodule-specific subtilisin-like proteases that have been linked to the infection process in actinorhizal Fagales, were also upregulated in the nodules of *D. glomerata* and *C. thyrsiflorus*. Nodule-specific defensin genes known from actinorhizal Fagales and Cucurbitales, were also found in *C. thyrsiflorus.* In summary, the results underline the variability of nodule metabolism in different groups of symbiotic plants while pointing at conserved features involved in the infection process.

## Introduction

Nitrogen is the element that most often limits plant growth. Members of four different plant orders can form root nodule symbioses with nitrogen-fixing soil bacteria (Mylona et al., 1995). In the root nodules, bacteria fix ambient dinitrogen while being hosted within plant cells. There are two types of root nodule symbioses; (i) most legume species and the non-legume genus *Parasponia* (Cannabaceae, Rosales) interact with a polyphyletic group of Gram-negative proteobacteria collectively known as rhizobia and (ii) actinorhizal plants interact with Gram-positive actinobacteria from the genus *Frankia*. The latter encompass 24 genera distributed over eight families across three orders: Cucurbitales (Datiscaceae and Coriariaceae), Fagales (Betulaceae, Casuarinaceae, and Myricaceae), and Rosales (Elaeagnaceae, Rhamnaceae, and Rosaceae) (Soltis et al., 1995; Pawlowski and Demchenko, 2012). Together with Fabales, they form a monophyletic group within the Fabid clade (Soltis et al., 1995; Angiosperm Phylogeny Group, 2016). Despite sharing a relatively recent ancestor (ca. 100 mya; Bell et al., 2010), actinorhizal species show high diversity in nodule anatomy, physiology, and metabolism (Swensen, 1996; Pawlowski and Demchenko, 2012).

Phylogenetic analyses have shown that symbiotic *Frankia* strains comprise three clusters that mostly correlate with host specificity (Normand et al., 1996; Sen et al., 2014). Cluster I strains nodulate members of three families in the Fagales [Betulaceae, Casuarinaceae (except for the genus *Gymnostoma*), and Myricaceae (except for the genus *Morella*)]. Cluster II strains display a wide host range nodulating all actinorhizal Cucurbitales and members of two Rosales families (Rosaceae and the genus *Ceanothus* in the Rhamnaceae). Cluster III strains nodulate species in Rosales (Elaeagnaceae and Rhamnaceae, except for the genus *Ceanothus*), and Fagales (the genera *Gymnostoma* and *Morella*). Cluster II, representing the earliest divergent clade within *Frankia*, comprises strains of which so far only two have been cultured (Gtari et al., 2015; Persson et al., 2015; Nguyen et al., 2016; Gueddou et al., 2018). This study focuses on two host plant species of cluster II *Frankia* strains, one from the Cucurbitales (*Datisca glomerata*, Datiscaceae) and one from the Rosales (*Ceanothus thyrsiflorus*, Rhamnaceae).

Several aspects of root nodule symbioses well researched in legumes are still understudied in actinorhizal plants. For example, nodule organogenesis and uptake of bacteria by plant roots are the result of the exchange of diffusible signals between host and bacteria (Oldroyd, 2013). Rhizobia signal to their host plants *via* lipochitooligosaccharide (LCO) Nod factors. In rhizobia, the synthesis of the LCO common backbone requires the enzymes encoded by the canonical *nod* genes *nodABC*. Plants perceive these LCO Nod factors by a heterodimer of LysM receptors, which then signal *via* the common symbiotic pathway that is shared with, and recruited from, arbuscular mycorrhizal symbioses (Oldroyd, 2013). Signaling in actinorhizal symbioses is less well examined. *Frankia* genome analysis led to the assumption that genes from clusters I and III do not signal via LCO Nod factors (Normand et al., 2007). Nevertheless, recent sequencing of *Frankia* cluster II genomes showed that they contain the canonical *nod* genes *nodABC*, and these genes are expressed *in planta* (Persson et al., 2015; Nguyen et al., 2016). So the first question concerns the presence and expression of genes orthologous to the known legume Nod factor receptor genes in roots and nodules of both plant species.

The second question to address is the mechanism of stable intracellular accommodation of *Frankia* within nodule cells. Cytological analysis of *D. glomerata* as representative of actinorhizal Cucurbitales have shown that the infection thread growth mechanism seems to differ from that used in Fagales and from that of Rosales (Berg et al., 1999; reviewed by Pawlowski and Demchenko, 2012). Genes encoding products relevant for infection thread growth have been identified in legumes (Suzaki et al., 2015), and the expression of their homologs could be examined in both actinorhizal species.

The third question concerns the nitrogen metabolism in nodules, specifically, the nitrogen source exported by the intracellular nitrogen-fixing microsymbionts to the plant cytoplasm. In legumes, it is generally accepted that this nitrogen source is ammonia, which is then assimilated in the cytosol of the infected nodule cells *via* the glutamine synthetase (GS)/glutamate synthase (GOGAT) pathway. High levels of plant GS expression in infected nodule cells have been shown for various legumes, such as *Phaseolus vulgaris* (Forde et al., 1989), *Glycine max* (Miao et al., 1991), and *Medicago sativa* (Temple et al., 1995). In similar fashion, ammonia has also been shown to represent the probable export product of *Frankia* in the actinorhizal tree *Alnus glutinosa* (Betulaceae, Fagales) based on the localization of plant GS (Hirel et al., 1982; Guan et al., 1997) and on the low levels of expression of *Frankia GS* in symbiosis (Alloisio et al., 2010). However, results of Berry et al. (2004, 2011) indicate that in *D. glomerata* nodules, *Frankia* exports an assimilated form of nitrogen, probably arginine, which is broken down in uninfected, rather than infected nodule cells, whereupon the ammonia is reassimilated in the GS/GOGAT pathway. Accordingly, plant GS expression is enhanced in nodules compared to roots, but not in infected cells (Berry et al., 2004). Thus, it remains unclear whether the export of an assimilated form of nitrogen from *Frankia* rather than ammonia is a feature of the nodules of actinorhizal Cucurbitales, or a feature of nodules induced by cluster II *Frankia* strains. Furthermore, the principal form of nitrogen transported in the xylem, and thus the N metabolite pattern in nodules, differs among actinorhizal plants. In actinorhizal Rosales, asparagine was identified as the principal xylem amino acid in *Hippophae, Elaeagnus, Ceanothus*, and *Discaria* (Schubert, 1986; Valverde and Wall, 2003), while in *D. glomerata*, glutamine, and glutamate were identified as the major xylem nitrogen transport forms (Berry et al., 2004, 2011; Persson et al., 2016). In actinorhizal Fagales (*Alnus* and *Casuarina*), the ureide citrullin was identified as the principal xylem nitrogen transport form (Walsh et al., 1984; Schubert, 1986).

The fourth question concerns the side effects of the oxygen protection system for nitrogenase, namely, oxidative and nitrosative stress. The enzyme complex nitrogenase is irreversibly deactivated by oxygen (Shah and Brill, 1973); however, the high energy demands of nitrogen fixation require optimal respiratory activity. In rhizobial symbioses the host reconciles this conflict; i.e., an oxygen barrier in the nodule cortex results in microaerobic conditions in the area of infected cells, in which oxygen carriers (class II hemoglobins, “leghemoglobins,” in legumes, and a class I hemoglobin in *Parasponia* spp.) enable optimal respiration (Minchin, 1997; Garrocho-Villegas et al., 2007). In contrast with rhizobia, *Frankia* strains can provide oxygen protection for nitrogenase themselves by differentiating specialized cell types, vesicles, surrounded by multi-layered envelopes containing hopanoids, bacterial steroid lipids. In these vesicles, nitrogenase is protected from oxygen (Parsons et al., 1987; Berry et al., 1993). As in actinorhizal nodules both host and bacteria can contribute to oxygen protection, the corresponding processes are diverse (Silvester et al., 1990). The process established in legumes leads to the production of high amounts of reactive oxygen species (ROS), first as a side product of leghemoglobin activity (Becana et al., 2000; Ott et al., 2005; Günther et al., 2007) and second as a side effect of mitochondrial respiration under hypoxia (Fukao and Bailey-Serres, 2004). This oxidative stress requires efficient detoxification. Furthermore, nodules are also exposed to nitrosative stress since nitric oxide (NO) is produced throughout the symbiosis in nodules of legumes (Hichri et al., 2015) and also in those of *Alnus firma* (Sasakura et al., 2006). NO can be synthesized by NO synthase, arise from non-enzymatic conversion of nitrite to NO in the apoplast (Bethke et al., 2004), or be produced by reductive pathways involving nitrate reductase (NR) or nitrite:NO reductase (Gupta and Igamberdiev, 2011). Detoxification of NO can be performed by class I hemoglobins (Igamberdiev et al., 2006; Perazzolli et al., 2006) and also by truncated hemoglobins (Sanz-Luque et al., 2015).

In nodules of *Ceanothus* spp., spherical vesicles resemble those found in *Frankia* liquid culture (Strand and Laetsch, 1977). Thus, similar to nodules of *A. incana* ssp. *rugosa*, the plant does not seem to contribute to prevent nitrogenase denaturation from oxygen (Silvester et al., 1988; Kleemann et al., 1994). In contrast, in nodules of *D. glomerata*, lanceolate *Frankia* vesicles arranged in radial orientation form a sphere around the central vacuole of the infected cells. As a second barrier at the place of oxygen access to this sphere, a thick layer of blanket mitochondria provides physiological oxygen protection (Silvester et al., 1999). The expression of a truncated hemoglobin in these cells indicates nitrosative stress (Pawlowski et al., 2007; Sanz-Luque et al., 2015).

Another question concerns the role of vitamins. The induction of genes encoding enzymes involved in thiamine (vitamin B1) biosynthesis has long been known for nodules of actinorhizal Fagales (Ribeiro et al., 1996; Hocher et al., 2011) and recently was also reported for the model legume *Lotus japonicus* (Nagae et al., 2016a). Despite its increasing interest, the role of thiamine in root nodule symbioses has yet to be understood. However, some data are available; e.g., the loss of function for a key gene involved on its biosynthesis has led to reduced nodule number in *L. japonicus* (Nagae et al., 2016a,b). Thus, nodule function in legumes and actinorhizal Fagales seems to require high amounts of thiamine. In this context, it is interesting that in *Arabidopsis thaliana* thiamine participates in the adaptation to oxidative (Tunc-Ozdemir et al., 2009) as well as to abiotic stresses (Ribeiro et al., 2005; Rapala-Kozik et al., 2012). However, it remains unclear whether the requirement for increased thiamine biosynthesis also extends to nodules of actinorhizal Cucurbitales and Rosales. The synthesis of folic acid (vitamin B9) which modulates root architecture (Ayala-Rodríguez et al., 2017) was included in the analysis.

Light-induced gene expression is not expected in subterranean plant organs, so the transcription of the plastidic gene encoding ribulose-1,5-bisphosphate carboxylase/oxygenase (RuBisCO) and the nuclear gene encoding RuBisCO activase in nodules of *D. glomerata* was a surprising result (*rbcL* and *rca;* Okubara et al., 1999). Yet, plants contain a variety of photoreceptors – phytochromes, cryptochromes and phototropins – to respond to light of different wavelengths, and these photoreceptors are not restricted to aerial parts, they also occur in roots (van Gelderen et al., 2018). Root development including nodulation is affected by light (Gundel et al., 2014; Shimomura et al., 2016). Moreover, some photoreceptors integrate light- and temperature signals (Casal and Qüesta, 2018). That being said, it is generally accepted that photosynthesis does not take place in subterranean organs, so the expression of *rbcL* and *rca* in *D. glomerata* nodules remained unexplained. Therefore, the analysis of expression of photosynthesis-related genes in nodules vs. roots was included in this study.

Subtilases are involved in many signaling pathways, e.g., pathways associated with organogenesis and senescence (Taylor and Qiu, 2017). In particular, subtilases are induced in plant-microbe interactions, e.g., arbuscular mycorrhiza (Takeda et al., 2009). In actinorhizal symbioses, nodule-specific subtilases were identified in Fagales (*A. glutinosa*; Ribeiro et al., 1995; *Casuarina glauca*; Laplaze et al., 2000) and recently also in Rosales (*Discaria trinervis*; Fournier et al., 2018). Therefore, their presence was also examined for nodules of *D. glomerata* and *C. thyrsiflorus.*

A mutualistic interaction has to guard against parasitic individuals that extract benefits without paying costs (“cheaters”) and thus proliferate more efficiently than their mutualistic brethren. In natural environments, many rhizobia have very low nitrogen fixation capacity (Oono et al., 2009). Plants from two groups of legumes, namely the Dalbergioids and the Inverted Repeat-Lacking Clade (IRLC), form cysteine-rich peptides (NCRs) in nodules which affect the differentiation of the microsymbionts. Their effects include amplification of the rhizobial genome, inhibition of rhizobial cell division, rhizobial cell elongation and -branching, as well as modification of the rhizobial plasma membrane (Van de Velde et al., 2010), a mechanism originally thought to have evolved for the control of rhizobial “cheaters.” However, recent results show that NCRs can have positive as well as negative effects on rhizobia (Pan and Wang, 2017). Nodule-specific expression of cysteine-rich peptides – in this case, defensins – was also found for actinorhizal Fagales (*A. glutinosa;* Carro et al., 2015) and of Cucurbitales (*D. glomerata*; Demina et al., 2013). So the question remains whether other actinorhizal plants, especially from the Rosales, form NCRs.

In summary, in this study, transcriptome sequencing combined with Reverse Transcription – quantitative PCR (RT-qPCR) was employed to compare nodules induced by *Frankia* cluster II strains on *D. glomerata* (Cucurbitales) and *C. thyrsiflorus* (Rosales). We addressed the following research questions: (1) Does expression of legume LCO Nod factor receptor orthologs support their role in the perception of bacterial signal factors in both host plant species? (2) Is the expression of homologs of legume genes encoding proteins required for infection thread formation induced in nodules compared to roots? (3) What can the differential expression of genes encoding enzymes in plant nitrogen metabolism tell us about the nitrogen source exported by the intracellular nitrogen-fixing microsymbionts to the plant cytoplasm? (4) What can the expression of genes encoding enzymes of the antioxidant defense system and of globins involved in NO detoxification tell us about the oxidative and nitrosative stress associated with the different oxygen protection systems for nitrogenase realized in both types of nodules? (5) Are genes involved in thiamine and/or folate biosynthesis induced in either type of nodule? (6) Are photosynthesis-associated genes expressed in both types of nodules? (7) Are subtilases expressed in both types of nodules, and are these subtilases orthologs of the nodule-specific subtilases identified for *A. glutinosa, C. glauca*, and *D. trinervis?* (8) Are nodule-specific cysteine rich peptides (defensins) expressed in nodules of *C. thyrsiflorus*, i.e., in a representative of the actinorhizal Rosales? Contributions to answering these questions will shed light on the commonalities and differences in root nodule symbiosis in relation to plant phylogeny.

## Materials and Methods

### Plant Material and Growth Conditions

*Datisca glomerata* seedlings were grown in a greenhouse under a photoperiod of 14 h light/8 h dark cycle with 22°C/19°C, respectively, and a relative humidity of 70%. They were fertilized with 1/4 strength Hoagland’s solution with 10 mM nitrogen (Hoagland and Arnon, 1938). 8-Week-old seedlings were inoculated with ground nodules of *D. glomerata* infected with *Candidatus* Frankia datiscae Dg1. After inoculation, plants were supplied with 1/4 strength Hoagland’s solution without nitrogen. Whole root nodules were harvested 12 weeks post inoculation.

*Ceanothus thyrsiflorus* plants were purchased from a nursery, Corn Flower Farm (Elkgrove, CA, United States) as cuttings, and were grown in a greenhouse in University of California, Davis. For successful nodulation, the plants were repotted into new medium (UC mix/perlite 1:1) to remove any fertilizer given to the plants by the nursery. Nodulation status of each plant was checked at this point to ensure that no plants were nodulated prior to any further treatment. After 4 weeks, the plants were inoculated using nodules from *C. thyrsiflorus* that had been inoculated with soil collected in Sagehen Experimental Forest (Truckee, CA, United States). Plants were maintained in a greenhouse and watered with deionized water and Hoagland’s solution without nitrogen. Plants were kept under natural daylight except during winter when they were kept under extended artificial daylight. The young (non-lignified) parts of nodules were cut off with a scalpel and flash frozen in liquid nitrogen.

### Preparation of RNA-Seq Libraries From *D. glomerata* and *C. thyrsiflorus* Nodules

Total RNA was isolated from five (*D. glomerata*) and three (*C. thyrsiflorus*) independent nodules as described previously (Demina et al., 2013). RNA-Seq libraries were prepared in strand-specific mode and sequenced with an Illumina HiSeq2500 platform (Illumina) yielding 256,841,770 (*D. glomerata;* detailed in Supplementary Table S1) and 157,205,694 (*C. thyrsiflorus*) paired-end reads (100 nt each). For *C. thyrsiflorus* an extra library was prepared and sequenced with an Illumina MiSeq instrument (Illumina). This new library was strand-specific, poly(A)-enriched and yielded a total of 36,740,942 (300 nt) paired-end reads that were exclusively used for assembly.

Raw data have been archived in BioProject PRJNA454374 (*D. glomerata*) and BioProject PRJNA454377 (*C. thyrsiflorus*).

### Filtering, Trimming and *de novo* Transcriptome Assemblies

For both species, *de novo* assemblies were generated following the same approach. Prokaryotic sequences were removed by mapping the raw reads against the genome of *Candidatus* Frankia datiscae Dg1 (GenBank accession NC_015656.1) using TopHat 2.0.12 (Trapnell et al., 2009). The quality of the retained reads was evaluated by FastQC 0.10.1 (Andrews, 2010). Illumina sequencing adapters and low quality reads were removed by Fastq-mcf 1.04.636 (Aronesty, 2013); next, low quality bases were trimmed at the 3’-end (10 nt) by Seqtk^1^. The resulting quality-filtered dataset, i.e., 200,795,069 paired-end reads (ca. 100 nt) for *D. glomerata* (summarized in Supplementary Table S1) and 36,589,680 paired-end reads (ca. 290 nt) for *C. thyrsiflorus*, was assembled with Trinity version r20140717 using default parameters (-K 25, -L 25; Grabherr et al., 2011).

For both species, the completeness of the transcripts in terms of expected orthology was evaluated by BUSCO 2.0.1 (Simão et al., 2015) against the reference plant dataset, embryophyta_odb9, which contained 1440 protein sequences and orthogroup annotations for major clades.

The *D. glomerata* Transcriptome Shotgun Assembly project has been deposited at DDBJ/EMBL/GenBank under the accession GGXR00000000. The version described in this paper is the first version, GGXR01000000. The *C. thyrsiflorus* Transcriptome Shotgun Assembly project has been deposited at DDBJ/EMBL/GenBank under the accession GGXO00000000. The version described in this paper is the first version, GGXO01000000.

### Transcriptome Functional Annotation

The *de novo* assembled transcriptomes were functionally annotated by similarity sequence search using Trinotate (Haas et al., 2013), yielding Gene Ontology terms, PFAM annotations and Enzyme commission (EC) numbers. Gene Ontology terms were assigned by Blast+ algorithms (Altschul et al., 1990) targeting the SwissProt non-redundant database. The longest ORFs were queried at the PFAM database (Punta et al., 2012) enabling domain predictions (HMMER 3.1b1; Finn et al., 2011). Additionally, longest ORFs were queried for signal peptides (SignalP 4.1; Petersen et al., 2011) and transmembrane domains (tmHMM 2.0c; Krogh et al., 2001). Trinotate is inclusive in the sense that it allows multiple PFAM entries to be assigned to a single contig. For simplicity, only the PFAM hit with lowest E-value was retained for each transcript.

### Expression Profiling

The transcript abundance was estimated by RSEM (Li and Dewey, 2011), available as a Perl script in the Trinity suite (version r20140717; Grabherr et al., 2011). For both species, the raw reads prepared in HiSeq2500 mode were mapped against the assembled contigs and both the fragment per kilobase of transcript per million reads (FPKM) and transcripts per million reads (TPM) were inferred for each transcript.

To assess differential expression, the contribution of different contigs was collapsed in single PFAMs by the sum of their FPKM. Every PFAM entry was further normalized against the abundance of the housekeeping control elongation factor 1 alpha (EF-1α; PF03143.12) from which the mean and log_2_[FC] was calculated in each library. These steps were carried out in RStudio (RStudio Team, 2015). EC numbers were used to graphically visualize MetaCyc metabolic pathways (Paley and Karp, 2006; Caspi et al., 2016). Functional annotation of transcriptomes and their expression profiles are accessible at doi: 10.17045/sthlmuni.6181763.v1.

### Real-Time Quantitative RT-PCR

In order to validate the RNA-Seq measurements, differential expression in roots vs. nodules was assessed by RT-qPCR. The method used was essentially as described in Zdyb et al. (2018). Primers were designed by Primer3 at NCBI Primer-Blast server and are listed in Supplementary Table S2 (*D. glomerata*) and Supplementary Table S3 (*C. thyrsiflorus*). Roots and nodules were harvested in liquid nitrogen and tissues were ground using pestle and mortar. Macerated tissues were immediately used for total RNA isolation using an on-column DNase treatment (Spectrum Total RNA isolation kit, Sigma-Aldrich, Germany). Prior to cDNA synthesis, 1 μg of total RNA was treated with RNase-free DNase using the Heat&Run kit (ArcticZymes, Norway). Total RNA was reverse transcribed in a final volume of 20 μl following the instructions of the TATAA GrandScript cDNA synthesis kit (TATAA Biocenter, Sweden); cDNA preparations were 10^-1^ diluted and 2 μl were used as a template in 10 μl PCR reactions; reactions were performed with 1x Maxima SYBR green (Thermo Fisher Scientific, Lithuania) and 300 nM of each primer in an Eco Real Time PCR System (Illumina, United StatesA); applied thermal conditions were 10 min 95°C for initial denaturation, extension at 60°C for 30 sec, 45 cycles. Melting dissociation curves were examined in order to circumvent the possibility of primer dimer. Demonstrative exponential phase Cq values of the housekeeping gene EF-1α were used to calculate normalization factors. Statistics were based in a balanced assay; relative quantities were back-transformed to Cq values from which an unpaired 2-tail Student’s test was conducted; p-values were adjusted according to Benjamini—Hochberg false discovery rate FDR (Benjamini and Hochberg, 1995). Statistics were performed in RStudio (RStudio Team, 2015).

### Phylogenetic Analysis

In order to assess the phylogeny of LysM-type and LysM receptors in *D. glomerata* and *C. thyrsiflorus*, NFR1, NFR5, and EPR3 protein candidates were selected from the transcriptomes by reverse Blast (Altschul et al., 1990). Blasted queries were selected based on previous reverse genetics studies involving the model legume *L. japonicus* (Radutoiu et al., 2003; Kawaharada et al., 2015). Individual candidate protein sequences were blasted against selected taxa in the RefSeq database with an *E*-value cutoff of 1e-50. Selected target taxa are listed in Supplementary Table S4. Unique sequences from this set (*n* = 180) plus the *D. glomerata* and *C. thyrsiflorus* candidate sequences were aligned using Clustal Omega version 1.2.4 (Sievers et al., 2014). From the alignment, truncated sequences were removed. Well-aligned positions were selected with BMGE using the BLOSUM62 substitution matrix (Criscuolo and Gribaldo, 2010). Phylogenetic trees were estimated based on maximum likelihood using RAxML version 8.2.10 (Stamatakis, 2014) using the PROTGAMMAAUTO model and rapid bootstopping (autoMRE) (Pattengale et al., 2009). The full alignment and the maximum likelihood tree are accessible at doi: 10.17045/sthlmuni.6384200.v1.

To gain insight about the phylogeny of subtilases, candidate proteins from *D. glomerata* and *C. thyrsiflorus* were placed in the comprehensive phylogeny of Taylor and Qiu (2017). Sequences were aligned to the original alignment with Clustal Omega version 1.2.4 (Sievers et al., 2014) and placed in the original phylogeny using RAxML-EPA version 8.2.10 (Stamatakis, 2014).

## Results and Discussion

### Illumina Sequencing, *de novo* Transcriptome Assembly and Annotation

After Illumina sequencing and quality filtering stages, reads were combined in a 183.4 Mb, N50 = 1,850 with an average contig length of 1,026 nt (*D. glomerata*; Supplementary Figure S1A) and in a 105.0 Mb, N50 = 1,275 with an average contig length of 715, 48 nt (*C. thyrsiflorus*; Supplementary Figure S1B) Trinity assembly (Grabherr et al., 2011). The resulting assembly contained 95,749 Trinity “genes” from a total of 164,856 isoforms (*D. glomerata*) and 97,521 Trinity “genes” from a total of 135,576 isoforms (*C. thyrsiflorus*).

Quality and completeness evaluation of the assemblies showed that 1130 [779 singletons and 351 duplicates] (*D. glomerata*) and 1103 [838 singletons and 265 duplicates] (*C. thyrsiflorus*) complete genes were assessed from a total of 1440 groups searched. Yet, 170 genes were fragmented and 140 were missing (*D. glomerata*); 135 genes were fragmented and 202 were missing (*C. thyrsiflorus*). In terms of expected orthology 90% (*D. glomerata*) and 86% (*C. thyrsiflorus*) of the transcripts could be assigned to members of the BUSCO plant set (Simão et al., 2015).

In total, 35,815 *D. glomerata* (37%) and 49,142 *C. thyrsiflorus* (50%) unique Trinity “genes” were annotated with SwissProt identifiers. *Arabidopsis thaliana* provided the best scoring alignment for both species (Supplementary Figures S1C,D). Of the top 100 most abundant transcripts annotated by PFAM within the “biological process” category, 44% showed shared functions between species (*D. glomerata*, Supplementary Figure S2; *C. thyrsiflorus*, Supplementary Figure S3; for numeric detail refer to doi: 10.17045/sthlmuni.6181772.v1).

GenBank accession numbers of sequences of transcripts analyzed in this study, as well as E.C. numbers of encoded enzymes, are listed in Supplementary Table S5; all *p*-values for RT-qPCR analyses are given in Supplementary Table S6.

### Genes Encoding LysM Receptors Associated With Symbiotic Signaling in Model Legumes Are Upregulated in Nodules of *D. glomerata* and *C. thyrsiflorus*

**FIGURE 1:**
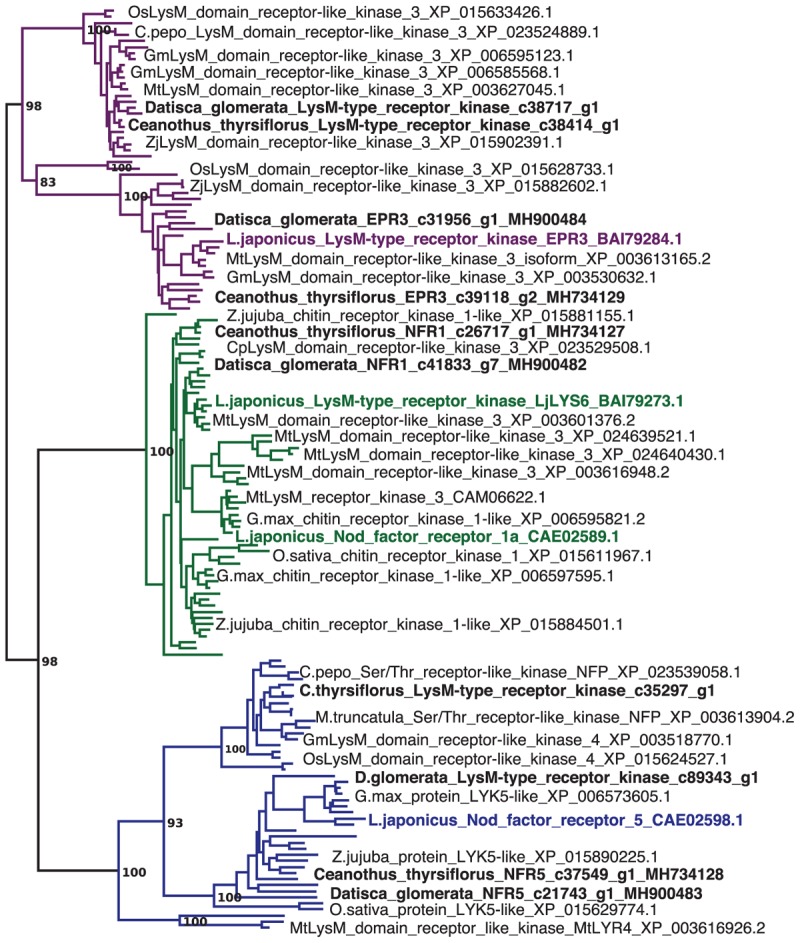
*Datisca glomerata* and *Ceanothus thyrsiflorus* nodules express putative orthologs of Nod factor receptors from legumes. Part of a maximum-likelihood phylogenetic reconstruction of LysM-type and LysM receptor kinases is shown (the full phylogenetic tree is shown in Supplementary Figure S10). Proteins encoded by genes identified based on studies of legume mutants are highlighted in color. Acronyms of included species are: Cp, *Cucurbita pepo*; Gm, *Glycine max*; Lj, *Lotus japonicus*; Mt, *Medicago truncatula*; Os, *Oryza sativa*; and Zj, *Ziziphus jujuba*. Different orthogroups are distinguished by color: EPR3 in purple, NFR1 in green, and NFR5 in blue. Candidate orthologs from *D. glomerata* and *C. thyrsiflorus* are given in black bold print. GenBank accessions are given.

**FIGURE 2:**
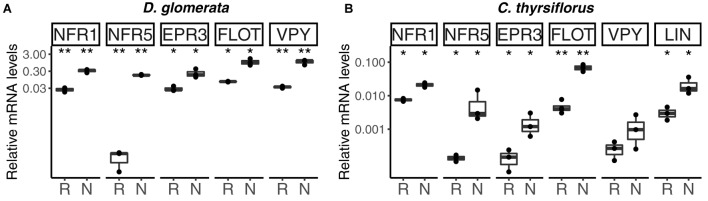
Expression profile of genes encoding orthologs of Nod factor receptors, and of genes encoding homologs of proteins linked to infection thread growth in legumes, in roots and nodules of *D. glomerata* **(A)** and *C. thyrsiflorus* **(B)**. Gene expression levels in roots (R) and nodules (N) are given relative to those of EF-1α. The median and interquartile range (IQR) of at least three biological replicates are shown. Differences between R and N are indicated at ^∗∗^*p* < 0.01, ^∗^*p* < 0.05 after student’s *t*-test followed by FDR multi comparison correction. *Y*-axis is given in log_10_ scale.

Sequences of nodulation-related genes identified based on legume mutants were used to identify putative orthologs in the nodule transcriptomes of both species. The phylogeny of LysM-type receptors and LysM receptor kinases, corresponding to NFR1 and NFR5 of the model legume *L. japonicus* (Radutoiu et al., 2003) supports the assignment of orthology (Figure 1). Reverse transcription – quantitative PCR (RT-qPCR) of these orthologs showed that in both *D. glomerata* and *C. thyrsiflorus*, these genes were expressed at significantly higher levels in nodules compared to roots (*p* < 0.01; Figure 2). While caution must be taken while interpreting these results, a role for these orthologs in an actinorhizal symbiosis with a Nod factor-producing *Frankia* strain may be suggested. Interestingly, in *C. glauca* (Casuarinaceae, Fagales), which is nodulated by a *Frankia* strain that does not contain the canonical *nod* genes, expression levels of the putative NFR1 ortholog do not differ between roots and nodules (Hocher et al., 2011). However, the expression of genes encoding putative Nod factor receptors was recently found to be induced in nodules of *Parasponia andersonii* compared to roots (van Velzen et al., 2018). It must be pointed out that since the *P. andersonii LjNFR5/MtNFP* ortholog is considered to have been recruited from arbuscular mycorrhizal (AM) symbiosis (Op den Camp et al., 2011; Miyata et al., 2016), these receptors might have additional functions that are responsible for the induction of their expression in nodules. Furthermore, transcriptional induction in nodules compared to roots was not found for the legume Nod factor receptors; expression of *L. japonicus NFR5* and of *Medicago truncatula NFP* is root specific, and expression levels of *L. japonicus NFR1* and its ortholog *M. truncatula LYK3* are similar in roots and nodules (Amor et al., 2003; Radutoiu et al., 2003; Smit et al., 2007). Hence, *D. glomerata* and *C. thyrsiflorus* orthologs may play a slightly different role in actinorhizal nodules. Still, differences in cell-specific distribution of Nod factor receptors between legumes and actinorhizal plants would not be surprising since the sizes of the corresponding gene families and degree of redundancy might conceivably differ between plant species. Altogether, the similarities with *Parasponia* regarding the differential expression of these genes, makes it very tempting to speculate about common signaling networks between non-legume hosts and Nod factor-producing bacteria (van Velzen et al., 2018). However, studies on gene knockouts are required to provide a definite answer.

### Genes Encoding Proteins Linked to Infection Thread Formation

In model legumes, several genes have been shown to encode proteins required for infection thread formation: a LysM receptor kinase, EPR3, that has been proposed to be involved in exopolysaccharide perception (Kawaharada et al., 2015), a flotillin that has been linked to infection thread growth (Haney and Long, 2010), as well as vapyrin (VPY; Murray et al., 2011) and LIN (Guan et al., 2013). These proteins are also required for AM symbioses. RT-qPCR analyses have shown that with the exception of LIN (Demina et al., 2013), the expression of the *D. glomerata* homologs of these genes was significantly enhanced in nodules compared to roots (for direct comparison with the *EPR3* and *flotillin* genes, the analysis of *VPY* and *LIN* was repeated here and included in Figure 2A). The corresponding homologs from *C. thyrsiflorus* were now analyzed by RT-qPCR; with the exception of VPY (*p* < 0.23), all these genes showed significant induction in nodules compared to roots (Figure 2). These data suggest that the corresponding proteins are required for infection thread growth in different nodulating lineages.

Expression of the putative *EPR3* ortholog genes was induced in nodules of both *D. glomerata* and *C. thyrsiflorus* (Figures 1, 2A,B). In legumes, the LysM receptor kinase EPR3 has been suggested to distinguish between compatible and incompatible rhizobial surfaces in order to abort infection threads containing incompatible rhizobia (Kawaharada et al., 2015). While the *EPR3* gene is not expressed in mature determinate nodules of *L. japonicus*, expression takes place in immature, i.e., non-nitrogen fixing nodules which would correlate with expression in the infection zone of indeterminate nodules (Kawaharada et al., 2017). Since actinorhizal nodules are indeterminate structures with a developmental gradient of infected cells, including a zone of infection, in the cortex (Ribeiro et al., 1995), nodule-enhanced expression of genes encoding proteins required in the infection zone would be expected. Yet, based on the results available so far, it is impossible to conclude whether the EPR3 orthologs of *D. glomerata* and *C. thyrsiflorus* are involved in *Frankia* surface recognition, or act as additional Nod factor receptors.

Similarly, the expression of the *flotillin* gene was induced in nodules compared to roots in both *D. glomerata* and *C. thyrsiflorus*. Flotillins have been linked to endocytosis and membrane shaping; they are targeted to membrane microdomains and have been shown to be involved in infection thread growth in legume nodules (Haney and Long, 2010). So this feature seems to be common in legume and actinorhizal nodules.

Vapyrin is required for epidermal penetration and infection thread development in AM symbioses (Pumplin et al., 2010). In legumes, vapyrin has been linked to the intracellular progression of infection threads and, consistent with this function, expression levels of *VPY* were found to be higher in nodules than in roots (Murray et al., 2011). Also in *D. glomerata*, *VPY* expression was induced in nodules (Demina et al., 2013). However, *VPY* expression was not induced in nodules of *C. thyrsiflorus.* This could be related to the fact that *C. thyrsiflorus* is infected intercellularly. *Frankia* hyphae colonize the apoplast and eventually are stably intracellularly accommodated in branching infection threads in infected cells. However, these infection threads do not show transcellular growth, in that infected cells are always infected from the apoplast, not from infection threads coming from an older infected cell (reviewed by Pawlowski and Demchenko, 2012). So it is possible that in actinorhizal symbioses, VPY is required only for transcellular growth of infection threads.

CERBERUS/LIN, an U-box protein containing WD40 repeats, is required for legume nodulation (Yano et al., 2009; Guan et al., 2013); for the *L. japonicus* ortholog (CERBERUS) also an effect on AM symbioses was shown. In *L. japonicus* mutants lacking this gene, the rhizobial infection process is aborted at a very early stage of infection thread formation, and elongation of intraradical hyphae of AM fungi is reduced (Takeda et al., 2013). While the expression of the corresponding gene is enhanced in nodules compared to roots of legumes, actinorhizal Fagales (*C. glauca*; Hocher et al., 2011) and of *C. thyrsiflorus* as representative of actinorhizal Rosales, this is not the case for *D. glomerata* (Datiscaceae, Cucurbitales; Demina et al., 2013). This result supports the hypothesis that a unique infection thread growth mechanism was established in actinorhizal Cucurbitales (Pawlowski and Demchenko, 2012).

### Genes Encoding Enzymes Involved in Arginine Metabolism Show Differential Expression Profiles in Nodules and Roots of *D. glomerata* and *C. thyrsiflorus*

Nodules of actinorhizal Cucurbitales have an unusual morphology in that infected cortical cells are not interspersed with uninfected cortical cells, but form a continuous patch, kidney-shaped in cross section, on one side of the acentric stele (Pawlowski and Demchenko, 2012). *D. glomerata* nodules have an unusual nitrogen assimilatory metabolism (Berry et al., 2011). Generally, in root nodule symbioses examined to date, the microsymbionts, whether rhizobia or Frankia, export the product of nitrogen fixation, ammonia, which is directly assimilated in the cytosol of the host infected cells via the glutamine synthetase (GS)/glutamate synthase (GOGAT) cycle (Supplementary Figure S4; Patriarca et al., 2002). In *D. glomerata*, however, cytosolic GS is expressed at high levels in the uninfected cells surrounding the patch of infected cells, but not in the infected tissue (Berry et al., 2004). Based on bacterial transcriptomics (Persson et al., 2015) and on the N metabolome of nodules (Berry et al., 2004), it was postulated that in *D. glomerata* nodules, symbiotic *Frankia* assimilates the ammonium produced by nitrogenase via bacterial GS/GOGAT, and exports an intermediate N storage product of the arginine cycle, presumably arginine, which is then broken down in the uninfected cells, leading to the re-assimilation of ammonium via the GS/GOGAT cycle, and, as demonstrated (Berry et al., 2004), the export of glutamine and glutamate from the nodule to the xylem stream.

**FIGURE 3:**
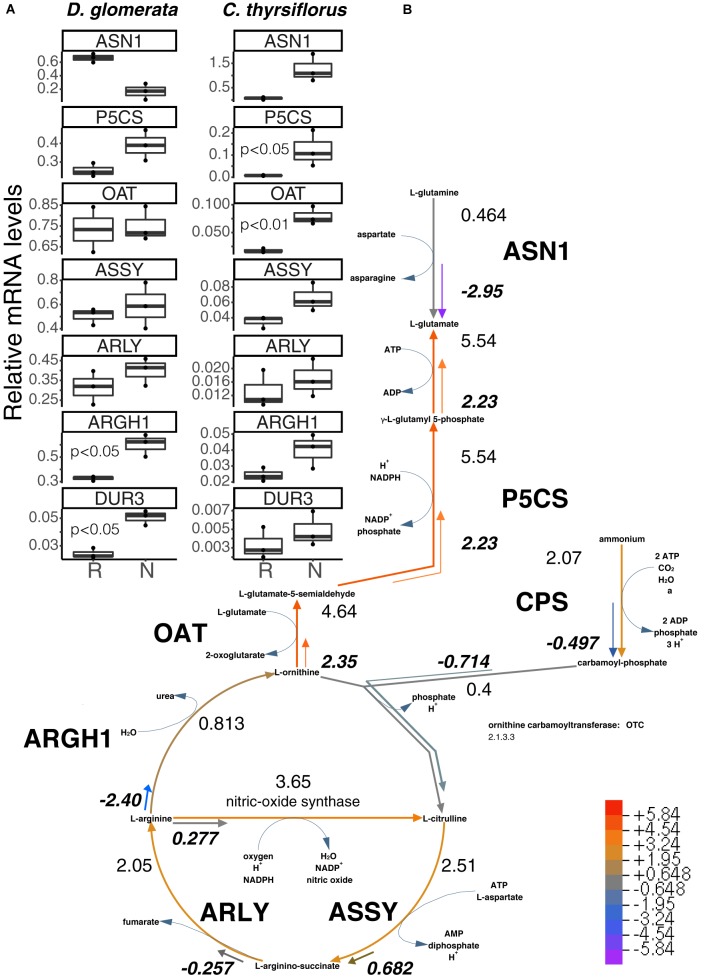
Superpathway for citrulline biosynthesis and link to the urea cycle. **(A)** Gene expression levels in roots (R) and nodules (N) were quantified by RT-qPCR for *D. glomerata* and *C. thyrsiflorus* and are relative to those of the housekeeping gene EF-1α. Median and IQR of three biological replicates are shown. Significant differences between R and N are indicated by *p*-values based on student’s *t*-test with FDR multi comparisons correction. *Y*-axis is given in log10 scale. **(B)** Gene expression levels are given in the context of the pathway, calculated as the log_2_[FC] relative to those of EF-1α estimated from the mean TPM of five (*D. glomerata*, long arrows) and three (*C. thyrsiflorus*, short arrows with values written in bold italics) independent RNA preparations. Explanatory heatmap is provided. Enzymes catalyzing these steps include: ASN1, asparagine synthase; P5CS, glutamate-5-semialdehyde dehydrogenase and glutamate-5-kinase (double function); CPS, ammonia-dependent carbamoyl phosphate synthetase; ASSY, argininosuccinate synthase; ARLY, argininosuccinate lyase; ARGH1, arginase; and OAT, ornithine aminotransferase. DUR3, high affinity plasma membrane urea transporter.

In our analysis, the genes encoding GS and GOGAT were upregulated in nodules vs. roots of *D. glomerata* (Supplementary Figure S4C), although the nodule/root ratio for GS expression was not significant due to the wide variation displayed between samples (*p* < 0.07). Expression levels of the gene encoding asparagine synthase (ASN1) showed a trend toward lower expression in nodules compared to roots (*p* < 0.11; Figure 3A). Pathways for arginine biosynthesis and degradation (Slocum, 2005; Winter et al., 2015) are depicted in Figure 3 and Supplementary Figure S5. Transcripts encoding several enzymes involved in these pathways were identified in both species and their expression was analyzed using RT-qPCR. Strikingly, a gene encoding an arginase homolog (ARGH1) which catalyzes the breakdown of arginine to urea and ornithine, was upregulated to very high levels in nodules of *D. glomerata* compared to roots (*p* < 0.027; Figure 3A). Furthermore, a gene encoding the homolog of the high affinity plasma membrane urea transporter DUR3 (Kojima et al., 2007) was identified and found to be induced in nodules compared to roots of *D. glomerata* (*p* < 0.05). Numbers of reads of urease transcripts were low, indicating that this function was not controlled on the transcriptional level (data not shown).

Genes encoding enzymes involved in the degradation of ornithine (Figure 3 and Supplementary Figure S5) were examined as well. A candidate for the mitochondrial isoform of *N*-acetylornithine aminotransferase (NAOAT), a close homolog of the aminotransferase class-III participating in the biosynthesis of citrulline in the actinorhizal tree *A. glutinosa* (Guan et al., 1997), was downregulated in *D. glomerata* nodules compared to roots (*p* < 0.05). However, no significant differences were found between the expression levels of the gene encoding the enzyme that catalyzes the transformation of L-ornithine to L-glutamate-5semialdehyde (ornithine aminotransferase, OAT; *p* < 0.55; Figure 3A) or for the gene encoding glutamate-5-semialdehyde dehydrogenase (P5CS; *p* < 0.074; Figure 3A).

Thus, the transcriptome data showed evidence for upregulation of arginine degradation in *D. glomerata* nodules. On the other hand, no evidence was found for the upregulation of arginine biosynthesis: the expression levels of the genes for argininosuccinate synthase (ASSY) and argininosuccinate lyase (ARLY) were similar in roots and nodules (Figure 3A).

In summary, the transcriptome data strongly support the role of arginine as intermediary nitrogen storage compound in root nodules of *D. glomerata* that is exported from *Frankia* and broken down by the plant in the uninfected cells. One breakdown product, urea, would be degraded to ammonium and CO_2_, with ammonium being reassimilated via the GS/GOGAT pathway, while the breakdown product ornithine could be used to form 2-oxoglutarate or another dicarboxylate, since carbon skeletons for ammonium assimilation have to be provided to *Frankia*. The upregulation of the glutamine-dependent carbamoyl phosphate synthetase (CPS; Supplementary Figure S5) in nodules compared to roots indicates that some of the reassimilated ammonium likely would go into plastidic arginine biosynthesis, either to replenish the arginine pool for protein biosynthesis, or as xylem transport form. The latter would be consistent with the observation that, in addition to the major xylem exports (glutamine and glutamate), low levels of arginine were detected in roots of nodulated plants of *D. glomerata* (Persson et al., 2016).

In *C. thyrsiflorus*, a different nitrogen assimilatory pattern emerges: expression of plant genes encoding GS and GOGAT was significantly induced in nodules vs. roots (Supplementary Figure S4C). Expression levels of the gene encoding asparagine synthase (ASN1) showed a trend toward elevated expression in nodules (*p* < 0.07), consistent with the fact that asparagine was reported as the major nodule amino acid in *Ceanothus* sp. (Schubert, 1986). In contrast with *D. glomerata*, expression of ARGH1 encoding arginase was not induced in nodules compared to roots (*p* < 0.11; Figure 3A), nor was expression of the gene encoding the high affinity plasma membrane urea transporter DUR3 (*p* < 0.36; Figure 3A). With regard to arginine biosynthesis, the gene encoding the glutamine-dependent CPS showed similar expression levels in nodules compared to roots (*p* < 0.93; Supplementary Figure S5), *ASSY* transcription was slightly elevated in nodules (*p* < 0.07), while *ARLY* showed similar expression levels in both organs (*p* < 0.45; Figure 3A). *NAOAT* showed a slight induction in nodules compared to roots (*p* < 0.056; Supplementary Figure S5C). By contrast, expression levels of *OAT* (*p* < 0.01; Figure 3A) and *P5CS* (*p* < 0.05; Figure 3A) were highly induced in *C. thyrsiflorus* nodules compared to roots (Figure 3A), suggesting the possibility of proline biosynthesis.

In summary, transcriptional analysis shows that plant N metabolism in nodules of *C. thyrsiflorus* (Rosales) is different from that in nodules of *D. glomerata*, even though both hosts are nodulated by closely related Cluster II *Frankia* strains. Thus, the specialization of N metabolism that occurs in *D. glomerata* is not necessitated by the capabilities of the microsymbiont, but shows the flexibility of metabolic adaptations possible in root nodules, in both the host and the microsymbiont.

### Hemoglobins in Nodules of *D. glomerata* and *C. thyrsiflorus*

No class II hemoglobin transcripts were identified in either transcriptome, suggesting that in neither system the buffering of free oxygen was required in the plant cytosol. Yet, in *Parasponia*, this function is performed by a class I hemoglobin (Sturms et al., 2010). In all cases, genes encoding the oxygen-buffering hemoglobin showed more than 1000-fold induction in nodules compared to roots.

**FIGURE 4:**
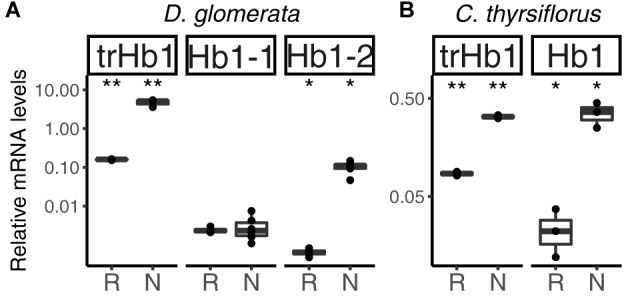
Hemoglobins in actinorhizal nodules of *D. glomerata* **(A)** and *C. thyrsiflorus* **(B)**. Transcript abundance was quantified by RT-qPCR. Gene expression levels in roots (R) and nodules (N) are relative to those of EF-1α. The median and IQR of at least three biological replicates are shown. Asterisks denote differences between R and N at ^∗∗^*p* < 0.01, ^∗^*p* < 0.05 by student’s *t*-test followed by FDR multi comparison correction. *Y*-axis is given in log_10_ scale.

Altogether, three hemoglobins were identified in the transcriptome of *D. glomerata*. Induction of the previously published (Pawlowski et al., 2007) truncated globin gene *trHB1* in nodules compared to roots was confirmed (*p* < 0.01; Figure 4A). Expression levels of a class I hemoglobin gene (Hb1-1) were also analyzed by RT-qPCR and shown to be similar in roots and nodules (Figure 4A). However, expression levels of a second class I hemoglobin gene (Hb1-2) were enhanced in nodules compared to roots (*p* < 0.05; Figure 4A). In a similar manner, the nodule transcriptome of *C. thyrsiflorus* contained transcripts of a truncated globin gene *trHB1*, whose expression levels were also markedly enhanced in nodules compared to roots (*p* < 0.01; Figure 4B). Expression of a class I hemoglobin gene was induced in nodules compared to roots (*p* < 0.05; *Hb1*; Figure 4B). In both species, the fold change of the class I hemoglobin gene in nodules was far below those found for oxygen-buffering hemoglobins.

In summary, among the set of genes encoding globins, only those associated with NO detoxification (one *trHb1*, one *Hb1* per species) were induced in nodules compared to roots in both systems. These results indicate that in both *D. glomerata* and *C. thyrsiflorus*, consistent with observations in actinorhizal Fagales (Heckmann et al., 2006; Sasakura et al., 2006), high activity toward NO detoxification is expected. However, despite the fact that hypoxia has also been previously associated with NO production in the model legume *M. truncatula* (Baudouin et al., 2006), and hypoxic conditions may prevail in the inner parts of infected cells of *D. glomerata* (Silvester et al., 1999), there is no evidence for differences in levels of nitrosative stress in nodules of *D. glomerata* vs. *C. thyrsiflorus* so far.

Under normoxic conditions, NO, an inhibitor of cytochrome c oxidase, can be oxidized to nitrite (Gupta and Igamberdiev, 2011). Nitrite is toxic and therefore needs to be rapidly catalyzed to ammonium by nitrite reductase NIR (Supplementary Figure S4B). Expression levels of *NIR* were repressed in nodules of *D. glomerata* (*p* < 0.02), but induced (*p* < 0.04) in those of *C. thyrsiflorus* (Supplementary Figure S4C). This difference could be explained by the hypothesis that oxidation of NO does not take place in infected cells of *D. glomerata* nodules since the high amounts of mitochondria (Silvester et al., 1999) lead to a reduction of the oxygen tension below normoxic conditions.

### Defense Against Reactive Oxygen Species (ROS) in Nodules of *D. glomerata* and *C. thyrsiflorus*

In many types of nitrogen-fixing root nodules, locally microaerobic conditions are established to protect the oxygen-sensitive nitrogenase enzyme complex (Jacobsen-Lyon et al., 1995). These conditions contribute to the production of reactive oxygen species (ROS; Fukao and Bailey-Serres, 2004). ROS can cause oxidative damage and therefore need to be quickly detoxified, a process that involves superoxide dismutase (SOD), peroxidases (PER) including ascorbate peroxidase (APX), and catalase (CATA).

**FIGURE 5:**
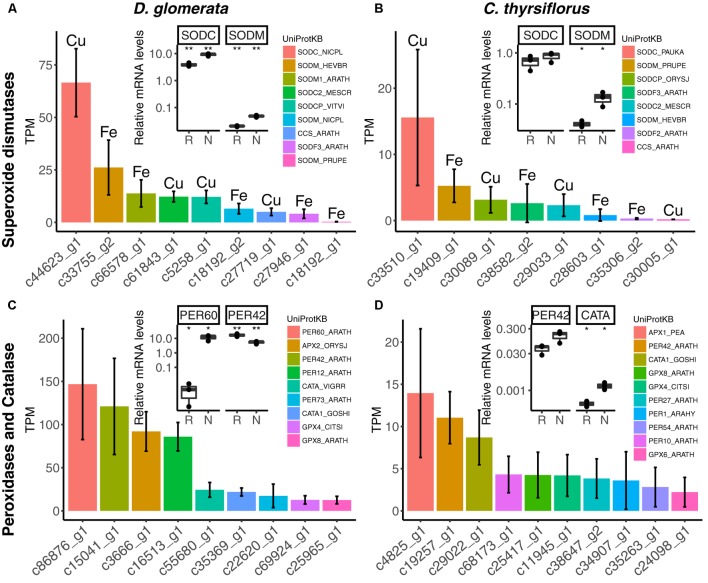
Expression of genes encoding superoxide dismutases, peroxidases and catalases in nodules of *D. glomerata* (left) and *C. thyrsiflorus* (right). Expression levels of superoxide dismutases **(A,B)**, peroxidases and catalases **(C,D)** are shown. Expression levels were estimated based on the mean TPM of five (*D. glomerata*) and three (*C. thyrsiflorus*) independent libraries. Bars were colored according to their annotated UniProt identifiers (see legend) and the cofactor used by each enzyme is given for **(A,B)**. Error bars show standard deviations. The relative abundance of mRNA in roots (R) and nodules (N) was quantified by RT-qPCR and is relative to that of the housekeeping gene EF-1α. The median and IQR of three biological replicates are shown. Asterisks highlight differences between organs at ^∗∗^*p* < 0.01, ^∗^*p* < 0.05 by student’s *t*-test followed by FDR multi comparison correction. *Y*-axis is given in log_10_ scale. UniProtKB homologs are indicated. After doi: 10.17045/sthlmuni.6181766.v1.

The nodule transcriptomes contained transcripts representing a number of *SOD* (Figures 5A,B) and *PER/CATA* genes (Figures 5C,D; listed in doi: 10.17045/sthlmuni.6181766.v1). Members displaying the highest TPM in each class were assayed by RT-qPCR. In *D. glomerata*, expression levels of two out of three *SOD* genes – encoding a copper-dependent SOD (SODC) and a manganese-dependent SOD (SODM) – were enhanced in nodules compared to roots (*p* < 0.01; Figure 5A). *PERs* displaying the highest transcriptome abundance (*PER60* and *PER42)* showed alternative regulation patterns based on RT-qPCR analysis: expression of *PER60* was strongly enhanced in nodules compared to roots, while *PER42* was repressed (*p* < 0.05 for both; Figure 5C).

For *C. thyrsiflorus*, the sub-classes of *SOD* genes with the highest transcriptome abundance were also encoding an SODC and an SODM (Figure 5B); only the expression of *SODM* was significantly induced in nodules compared to roots (*p* < 0.01; Figure 5B). Like in *D. glomerata, PER42* represented the most abundant *PER*; however, in *C. thyrsiflorus* its expression levels were not significantly increased in nodules compared to roots (*p* < 0.076; Figure 5D). On the other hand, albeit being expressed at relative extreme low levels, *CATA* was induced in nodules compared to roots (*p* < 0.05; Figure 5D).

**FIGURE 6:**
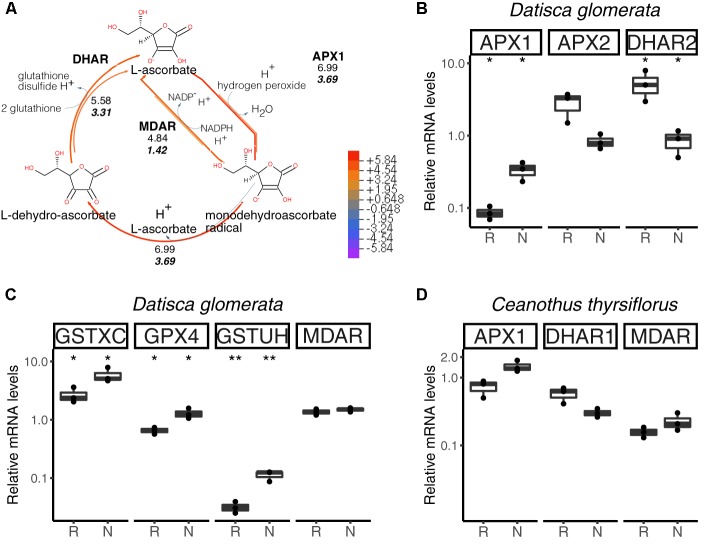
Hydrogen peroxide (H_2_O_2_) detoxification *via* the ascorbate/glutathione cycle. H_2_O_2_ is generated from the superoxide radical by superoxide dismutase (SOD; not shown). L-ascorbate serves as electron donor *via* ascorbate peroxidase (APX1, APX2). GPX4, glutathione peroxidase; GSTXC and GSTUH, glutathione *S*-transferases; MDAR, monodehydroascorbate reductase; DHAR, dehydroascorbate reductase. **(A)** Expression levels represented in the cycle were calculated as the log_2_[FC] relative to those of EF-1α estimated from the mean TPM of five (*D. glomerata*, long arrows) and three (*C. thyrsiflorus*, short arrows with values written in bold italics) independent libraries. Explanatory heatmap is provided. **(B–D)** Transcript abundance quantified by RT-qPCR for candidate genes in nodules of *D. glomerata* **(B,C)** and *C. thyrsiflorus* **(D)** is shown. Gene expression levels in roots (R) and nodules (N) are relative to those of the housekeeping control EF-1α. The median and IQR of three biological replicates are shown. Differences between R and N are highlighted at ^∗∗^*p* < 0.01, ^∗^*p* < 0.05 after student’s *t*-test with FDR multi comparison correction. *Y*-axis is given in log_10_ scale. Species names and UniProtKB homologs are indicated. After doi: 10.17045/sthlmuni.6181766.v1.

The main pathway for ROS scavenging in plants is the ascorbate-glutathione cycle (Figure 6A). In *D. glomerata*, expression assessment performed by RT-qPCR for members of this pathway showed that an L-ascorbate peroxidase 1 gene (*APX1*; Figure 6B), two glutathione *S*-transferase genes (*GSTXC* and *GSTUH*; Figure 6C), and a glutathione peroxidase gene (*GPX4*; Figure 6C) were induced in nodules compared to roots (*p* < 0.05). Expression of an *APX2* gene, the L-ascorbate peroxidase gene displaying the highest TPM, was slightly enhanced in roots compared to nodules (*p* = 0.054; Figure 6B); while the monodehydroascorbate reductase gene *MDAR* was expressed at similar levels in roots and nodules (Figure 6C).

For *C. thyrsiflorus*, expression levels of *APX1, DHAR1*, and *MDAR* were analyzed in roots vs. nodules. Although no significant differences were found, *APX1* expression was slightly enhanced in nodules (*p* = 0.060), while *DHAR1* expression was slightly enhanced in roots (*p* = 0.066) (Figure 6D). Additionally, the levels of three glutathione peroxidases genes (encoding the strongest homologs of GPX4, GPX6, and GPX8) were analyzed. Unlike in *D. glomerata*, all these genes were expressed at similar levels in roots and nodules (data not shown).

In summary, when comparing gene expression levels for components of the ascorbate/glutathione cycle, no significant differences were found between roots and nodules of *C. thyrsiflorus*; whilst, in *D. glomerata*, some genes encoding enzymes of these pathways were induced in nodules compared to roots. Although caution must be exercised while interpreting transcriptome data, these results suggest that ROS stress might be higher in nodules of *D. glomerata* than in those of *C. thyrsiflorus.* This is consistent with the assumption that in *D. glomerata* the plant participates in the protection of nitrogenase from oxygen, creating microaerobic conditions *via* a blanket of mitochondria (Silvester et al., 1999), and these conditions lead to enhanced ROS production (Fukao and Bailey-Serres, 2004). In *C. thyrsiflorus*, on the other hand, nitrogenase seems to be protected from oxygen due to the thick envelope surrounding spherical *Frankia* vesicles (Strand and Laetsch, 1977), similar to the situation in *Alnus* nodules (Kleemann et al., 1994), while normoxic conditions prevail in the plant cell. Thus, the induction of *NIR* expression in *C. thyrsiflorus* nodules must be due to nitrosative, and not oxidative stress.

### Key Genes Involved in the Biosynthesis of the Vitamins Thiamine and Folic Acid Are Differentially Expressed in Roots and Nodules of *D. glomerata* and *C. thyrsiflorus*

**FIGURE 7:**
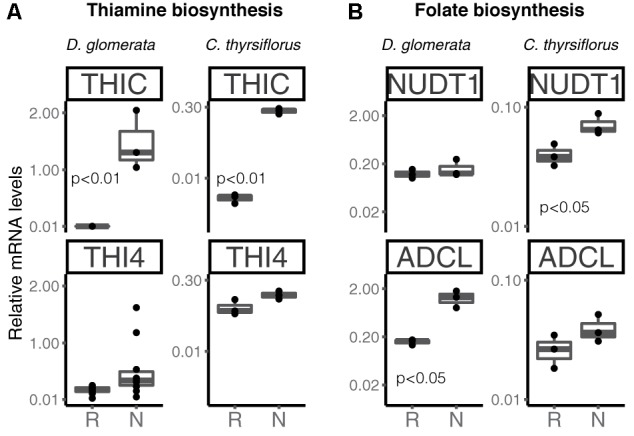
Expression of key genes encoding enzymes involved in the biosynthesis of the vitamins thiamine **(A)** and folic acid **(B)** in roots and nodules of *D. glomerata* and *C. thyrsiflorus*. Gene expression levels in roots (R) and nodules (N) are relative to those of EF-1α. THIC, phosphomethylpyrimidine synthase; THI4, bifunctional enzyme hydroxyethylthiazole kinase and thiamine-phosphate pyrophosphorylase; NUDT1, nudix hydrolase and ADCL, 4-amino deoxychorismate lyase. The median and IQR of at least three biological replicates are shown. Differences between R and N are indicated by *p*-values after student’s *t*-test followed by FDR multi comparison correction. *Y*-axis is given in log_10_ scale.

Transcripts of genes encoding members of the thiamine biosynthetic pathway were identified in nodules of *D. glomerata* (Supplementary Figure S6, long arrows) and in those of *C. thyrsiflorus* (Supplementary Figure S6, short arrows). Expression levels of the key genes *THIC* and *THI4* were quantified by RT-qPCR in roots and nodules of both species (Figure 7A). *THIC* showed strong induction in nodules compared to roots in both species (*p* < 0.01). Expression levels of THI4, the homolog of which is upregulated in nodules of *A. glutinosa* compared to roots (Ribeiro et al., 1996), showed slight upregulation in nodules of *D. glomerata* (*p* < 0.053), but not in those of *C. thyrsiflorus* (*p* < 0.1; Figure 7A).

Thus, like nodules of legumes and actinorhizal Fagales, nodules of actinorhizal Cucurbitales and Rosales require high amounts of thiamine, possibly due to its function in the oxidative stress response (Tunc-Ozdemir et al., 2009). Recently, it was shown that the AM fungus *Rhizophagus irregularis* lacks the toolkit for thiamine biosynthesis (Tisserant et al., 2013); spores of *R. irregularis* accumulated higher levels of thiamine than roots of *L. japonicus* during AM symbiosis (Nagae et al., 2016b). However, it is unlikely that symbiotic auxotrophy of symbiotic nitrogen-fixers, analogous for the symbiotic auxotrophy of rhizobia for branched chain amino acids (Prell et al., 2009), is the reason behind the upregulation of thiamine biosynthesis-related genes in nodules. No changes in gene expression levels were observed for *THIC in planta* vs. in N-replete cultures for the *A. glutinosa*-infective *Frankia* strain ACN14a (Alloisio et al., 2010); so there is no reason to assume that *Frankia* thiamine biosynthesis is shut down in symbiosis.

Transcripts of all members of the folic acid (vitamin B9) biosynthesis pathway were identified in nodules of both plant species (Hanson and Gregory, 2011; Supplementary Figure S7); RT-qPCR was performed for two key genes encoding nudix hydrolase (NUDT1) and 4-amino deoxychorismate lyase (ADCL) (Figure 7B). These enzymes map to different branches of the pathway; it should be pointed out that NUDT1 may have another function as well (Ogawa et al., 2005). For *D. glomerata*, *ADCL* was significantly induced in nodules compared to roots (Figure 7B); for *C. thyrsiflorus*, *NUDT1* was induced in nodules compared to roots, while *ADCL* was not (*p* = 0.2) (Figure 7B). This is, to our knowledge, the first evidence for a possible role of folic acid in actinorhizal nodules; further studies are required to elucidate its function.

### Photosynthesis-Related Genes Are Expressed in Nodules of *D. glomerata* and *C. thyrsiflorus*

**FIGURE 8:**
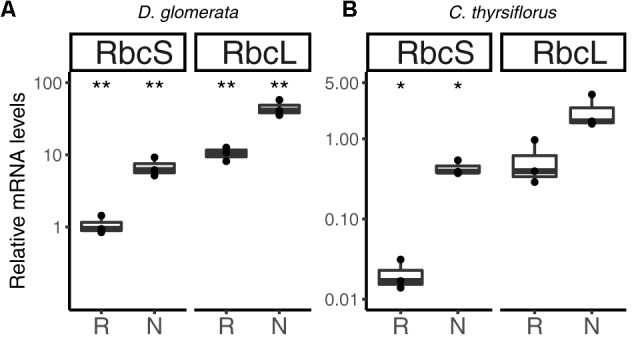
Relative expression levels from genes encoding components of the photosynthetic apparatus in subterranean organs of *D. glomerata* **(A)** and *C. thyrsiflorus* **(B)**. Results for the small (RbcS) and large (RbcL) subunit of RuBisCO are presented. Transcript abundance was quantified by RT-qPCR. Gene expression levels in roots (R) and nodules (N) are relative to those of the housekeeping gene EF-1α. The median and IQR of three biological replicates are shown. Differences between R and N are highlighted at ^∗∗^*p* < 0.01, ^∗^*p* < 0.05 after student’s *t*-test with FDR multi comparison correction. *Y*-axis is given in log_10_ scale.

Previous studies have shown that nitrogen-fixing nodules of *D. glomerata* express the gene encoding ribulose-1,5-bisphosphate carboxylase/oxygenase (RuBisCO) activase, an enzyme commonly associated with photosynthesis, and also the gene encoding the large subunit of RuBisCO (Okubara et al., 1999). The latter was confirmed in this study using RT-qPCR; transcription of both the nuclear gene for the small (RbcS) and the plastidic gene for the large (RbcL) subunit was highly induced in nodules compared to roots (*p* < 0.01; Figure 8A).

Similarly, RuBisCO activase transcripts were found in the nodule transcriptome of *C. thyrsiflorus* (data not shown). RT-qPCR analysis showed that also here, *RbcS* transcription was induced in nodules compared to roots (*p* < 0.05); instead, expression levels of *RbcL* were not significantly elevated in nodules compared to roots (*p* < 0.06) (Figure 8B).

Plants express their photoreceptors – phytochromes, cryptochromes and phototropins – not only in their aerial parts, but also in roots (Yokawa and Baluška, 2015; van Gelderen et al., 2018). Roots show negative phototropism in response to direct light, and can also react to stem-piped light. Consequently, light responses in subterranean organs might not be surprising, but the expression of genes encoding RuBisCO subunits is. Nevertheless, a non–Calvin cycle, i.e., a CO_2_-scavenging role for RuBisCO has been demonstrated for developing *Brassica napus* embryos (Schwender et al., 2004); so it is possible that RuBisCO performs a similar function in nodules.

### Expression of Several Serine Proteases Is Strongly Induced in Nodules Compared to Roots in Both Species

**FIGURE 9:**
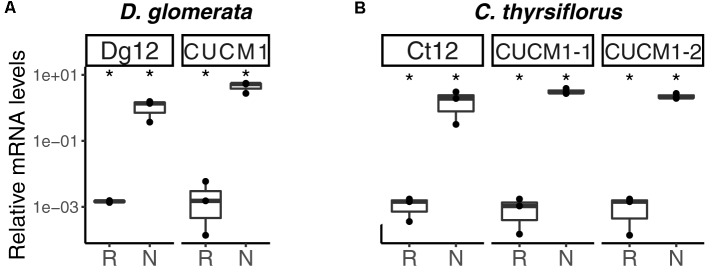
Subtilisin-type peptidases belonging to the S8 family are expressed in nodules of *D. glomerata* **(A)** and *C. thyrsiflorus* **(B)**. The transcript abundance in roots (R) and nodules (N) was quantified by RT-qPCR relative to that of the housekeeping gene EF-1α. The median and IQR of three biological replicates are shown. Differences between R and N are highlighted at ^∗^*p* < 0.05 after student’s *t*-test with FDR multi comparison correction. *Y*-axis is given in log_10_ scale.

Analysis of the *D. glomerata* nodule transcriptome revealed transcripts encoding 178 peptidases from 27 families. Subtilases represented 23.6% of this total, and from this group, transcripts encoding for members of the S8 family (subtilisin-like proteases) were the most abundant (42 contigs; Supplementary Figure S8A; Taylor and Qiu, 2017). Based on TPM data, the two S8 subtilases displaying the highest expression levels were selected for RT-qPCR analysis. Results showed high levels of induction in nodules compared to roots of the ortholog of *Ag12/Cg12/Dt12* (Ribeiro et al., 1995; Laplaze et al., 2000; Fournier et al., 2018), named *Dg12*, and of a gene encoding a cucumisin homolog (*CUCM1*; Figure 9A).

Global expression analysis of peptidases in *C. thyrsiflorus* nodules revealed major commonalities with that of *D. glomerata* nodules. Despite that, subtle differences could also be noticed. For instance, although fewer transcripts were annotated as peptidases (total = 147), they were actually more diverse occurring in 37 distinct families. In a similar manner, genes encoding serine proteases of the subtilisin S8 family were expressed at the highest levels (22 contigs, 15%; Supplementary Figure S8B). Those displaying the highest TPM values were selected for roots vs. nodules RT-qPCR analysis. In line with the results described for *D. glomerata*, expression of the homolog of *Ag12/Cg12/Dt12*, named *Ct12*, and of two cucumisin genes (*CUCM1-1*, transcript c29196_g1; *CUCM1-2*, transcript c34419_g1) was highly induced in nodules compared to roots (*p* < 0.05; Figure 9B). In addition, the expression levels of the genes encoding the homologs of the *Arabidopsis* subtilases AIR3 and SUBL were also analyzed, but they did not differ between roots and nodules (data not shown). These results are summarized for both species in doi: 10.17045/sthlmuni.6181760.v1.

**FIGURE 10:**
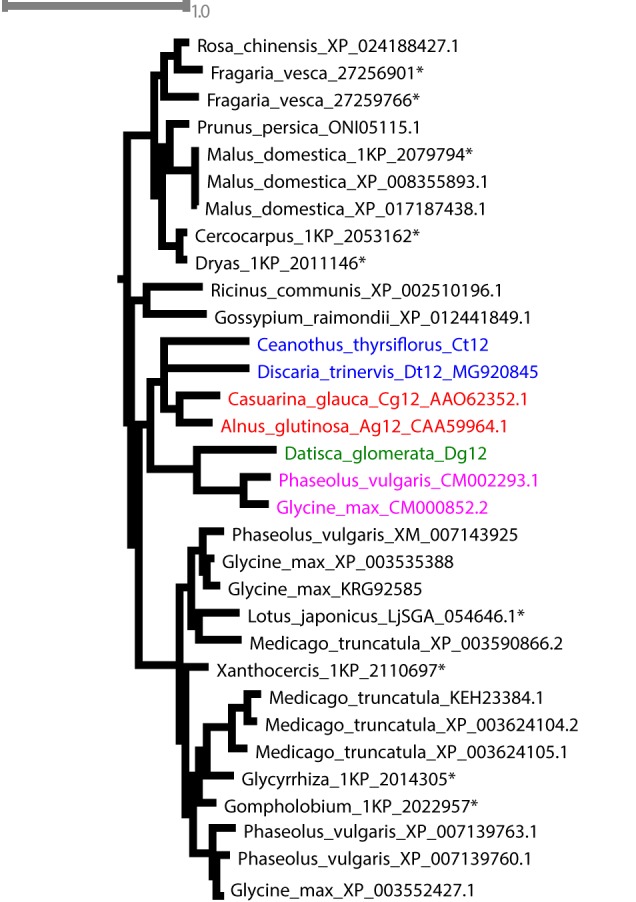
Maximum-likelihood phylogenetic tree of subtilases. Candidates from *D. glomerata* and *C. thyrsiflorus* (in bold) were analyzed for phylogenetic placement using the comprehensive dataset of Taylor and Qiu, 2017 (see section “Materials and Methods”). When possible, GenBank accession numbers are provided after the species name. Otherwise, an asterisk (^∗^) refers to the nomenclature used by Taylor and Qiu (2017). The analyzed candidates form a clade with proteins from Rosales (in blue), Dt12, *D. trinervis* (Fournier et al., 2018); Fagales (in red), Cg12, *Casuarina glauca* (Laplaze et al., 2000; Svistoonoff et al., 2003, 2004); Ag12, *A. glutinosa* (Ribeiro et al., 1995); and Fabales (in pink), CM002293.1 from *Phaseolus vulgaris* and CM000852.2 from *Glycine max*.

Phylogenetic analysis confirmed the orthology of Ag12, Cg12, Dt12, Dg12, and Ct12 (Figure 10) and of the nodule-enhanced cucumisins of *D. glomerata* and *C. thyrsiflorus*, respectively (Supplementary Figure S9). In summary, the extracellular subtilases whose expression is correlated with infection thread formation in intracellularly infected actinorhizal species (Ribeiro et al., 1995; Svistoonoff et al., 2003, 2004), and with intercellular infection foci in intercellularly infected actinorhizal species (Fournier et al., 2018) seem to have a common origin. Further studies will be required to determine whether the nodule-specific cucumisins are a feature of actinorhizal Cucurbitales, or of actinorhizal symbioses in general.

### Nodule-Specific Defensins: The Actinorhizal Equivalents of Legume Nodule Cysteine-Rich Peptides (NCR)

**FIGURE 11:**
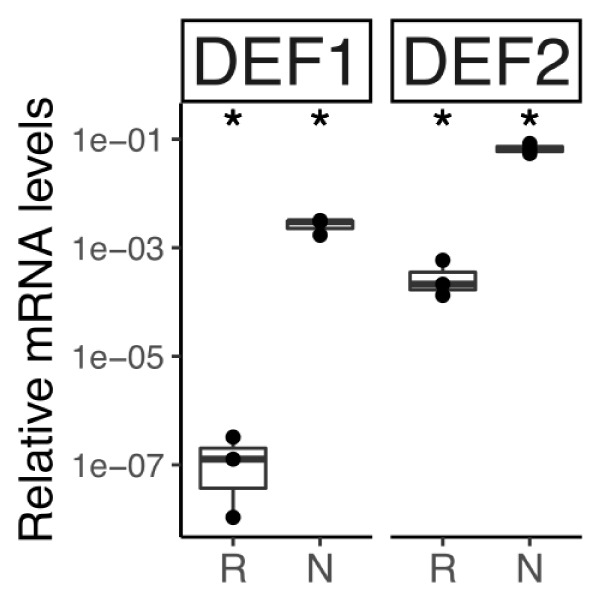
Relative transcript abundance of genes encoding defensin-like peptides in belowground organs of *C. thyrsiflorus*. Expression levels in roots (R) and nodules (N) are relative to those of the housekeeping gene EF-1α. The median and IQR of three biological replicates areshown. Differences between R and N are indicated for ^∗^*p* < 0.05 after student’s *t*-test followed by FDR multi comparison correction. *Y*-axis is given in log_10_ scale.

The transcriptome of *C. thyrsiflorus* was examined for genes encoding small cysteine-rich peptides. Two were identified and they represent class I defensins (Parisi et al., 2018). RT-qPCR analysis showed that both were strongly induced in nodules compared to roots (Figure 11).

While in legumes NCRs only occur in the IRLC clade and in the Dalbergioid lineage (Van de Velde et al., 2010; Czernic et al., 2015), in actinorhizal plants nodule-specific defensins have now been found across the three orders: Cucurbitales (Demina et al., 2013), Fagales (Carro et al., 2015), and Rosales (this study). Legume NCRs have been shown to affect bacteroid membrane permeability and induce polyploidy, turning the bacteroid state in a terminal differentiation (Alunni and Gourion, 2016). While the latter function has not yet been shown for actinorhizal defensins since analysis of polyploidy in a filamentous bacterium is a technical problem, *A. glutinosa* nodule-specific defensins have been demonstrated to affect membrane permeability of *Frankia alni* ACN14a for amino acids and to negatively affect viability of ACN14a *in vivo* (Carro et al., 2015). This might be interpreted to mean that in perennial nodulating plants (*D. glomerata* is biennial, and all other actinorhizal species are perennial) the defense against microbial “cheaters” is more urgent than in annual plants (most legumes analyzed in detail are annuals). Further studies are required to understand the distribution of antimicrobial peptides in actinorhizal vs. legume nodules.

## Conclusion

All host plants examined thus far of *Frankia* strains containing the canonical *nod* genes, contain orthologs of legume Nod factor receptors the expression of which is induced in nodules compared to roots.

Analysis of transcript levels in roots vs. nodules of genes encoding enzymes from arginine metabolism indicates that while arginine is the likely form of nitrogen exported by *Frankia* in nodules of *D. glomerata*, it does not seem to play this role in nodules of *C. thyrsiflorus.*

The oxygen protection system for nitrogenase realized in nodules of *D. glomerata* seems to lead to greater oxidative stress than the system realized in nodules of *C. thyrsiflorus.* However, the production of high levels of nitric oxide under normoxic conditions seems to lead to nitrite production in *C. thyrsiflorus* nodules, as indicated by the induction of nitrite reductase expression.

Thiamine biosynthesis is induced in actinorhizal nodules of all three different orders. Folic acid biosynthesis so far was only found to be induced in nodules of *D. glomerata.*

Nodule-specific subtilisin-like proteases that are involved in infection in actinorhizal nodules, seem to have a common evolutionary origin.

Nodule-specific defensins are found in actinorhizal species from all three different orders.

## Data Availability

The transcriptome raw data were submitted to GenBank as BioProjects (PRJNA454375 and PRJNA454377). Transcriptome Shotgun Assemblies were deposited at DDBJ/EMBL/GenBank under the accessions GGXR01000000 and GGXO01000000. All cDNA sequences analyzed by RT-qPCR were submitted to GenBank; a list of names and accession numbers is presented in Supplementary Table S5. The annotation lists are available on FigShare (references in the text).

## Author Contributions

MS and KP: conceptualization. MS, DL, TVN, RvV, and KP: methodology. MS, DL, RvV, and KB: investigation. MS, RvV, and DL: formal analysis. MS and DL: visualization. MS writing – original draft. MS, RvV, DL, KB, AB, and KP: writing – review and editing. KP: funding acquisition and supervision.

## Conflict of Interest Statement

The authors declare that the research was conducted in the absence of any commercial or financial relationships that could be construed as a potential conflict of interest.

## Footnotes

1 https://github.com/lh3/seqtk

## Supplementary Material

Figure S1 Statistics of Trinity assembly (upper panels) and Trinotate annotation (lower panels). Length distribution of assembled transcripts from nodules of *D. glomerata* **(A)** and *C. thyrsiflorus* **(B)** is shown. For *D. glomerata*, five independent libraries prepared on Illumina HiSeq 2500 technology were combined and processed on Trinity to generate an assembly with a median (N50) of 1,850 nt. *C. thyrsiflorus* assembly was generated from an Illumina MiSeq library (see section “Materials and Methods”); when combined, the processed reads yielded an assembly with a median (N50) of 1,275 nt. Axes are given in log_10_ scale. The frequency of hierarchical species assigned by Trinotate for *D. glomerata* **(C)** and *C. thyrsiflorus* **(D)** is based on the total number of BlastX hits retrieved. Legend: ARATH, *Arabidopsis thaliana*; TOBAC, *Nicotiana tabacum* (common tobacco)*;* ORYSJ, *Oryza sativa* ssp. *japonica* (rice)*;* HUMAN, *Homo sapiens* (human)*;* MOUSE, *Mus musculus* (mouse)*;* SCHPO, *Schizosaccharomyces pombe* (fission yeast)*;* DICDI, *Dictyostelium discoideum* (slime mold)*;* DROME, *Drosophila melanogaster* (Fruit fly)*;* YEAST, *Saccharomyces cerevisiae* (baker’s yeast). Species frequency is represented by log_10_ scale with actual numbers at the top of the bars.

Figure S2 Averaged Transcript per Million reads (TPM) covering the Top 100 contigs assigned within the “biological process” category of *D. glomerata*. Top hits were selected based on the mean TPM of five libraries. Plotted is the median and IQR across these libraries (after doi: 10.17045/sthlmuni.6181772.v1).

Figure S3 Averaged Transcript per Million reads (TPM) covering the top 100 contigs assigned within the “biological process” category of *C. thyrsiflorus*. Top hits were selected based on the mean TPM of three libraries. Plotted is the median and IQR across these libraries (after doi: 10.17045/sthlmuni.6181772.v1).

Figure S4 Routes for primary nitrogen fixation in plants include those of ammonia **(A)** and nitrate assimilation **(B)**. The assimilation of ammonia is catalyzed in two steps in the GS/GOGAT cycle. Ammonia is first bound to L-glutamate, yielding L-glutamine, by glutamine synthetase (GS). L-glutamine and 2-oxoglutarate serve as substrates for glutamate synthase (GOGAT) to yield two molecules of L-glutamate **(A)**. Alternatively, nitrate can be uptaken from the rhizosphere. In the cytosol, nitrate can be reduced to nitrite by nitrate reductase (NR) and converted to ammonia by nitrite reductase (NIR). The final step involves the incorporation of ammonia into glutamine by GS **(B)**. Expression levels in graphic pathways **(A,B)** were calculated as the log_2_[FC] relative to those of the housekeeper EF-1α estimated from the mean TPM of five (*D. glomerata*, long arrows with values written in normal font) and three (*C. thyrsiflorus*, short arrows with values written in bold italics) independent RNA preparations. Enzyme commission (EC) numbers and explanatory heatmap are provided. **(C)** Displays the transcript abundance quantified by RT-qPCR for key genes involved in the above mentioned pathways. Plotted results comparing the expression levels in roots (R) and nodules (N) are relative to those of the housekeeping gene EF-1α; the median and IQR of at least three biological replicates are given. Asterisks mark differences between roots and nodules for ^∗^*p* < 0.05 calculated by student’s *t*-test followed by Benjamini–Hochberg FDR correction. The *Y*-axis is given in log_10_ scale.

Figure S5 Arginine biosynthesis in chloroplasts *via* L-ornithine and its link to ammonium assimilation in the GS/GOGAT pathway. Ornithine synthesis starts from glutamate in a series of committed acetylation reactions (steps 1–5). Enzymes catalyzing these first steps include: (1) NAGS: *N*-acetylglutamate synthase; (2) NAGK: *N*-acetylglutamate kinase; (3) NAGPR: *N*-acetyl-glutamyl-*P*-reductase; (4) NAOAT: *N*-acetylornithine aminotransferase; and (5) NAOD: *N*-acetylornithine deacetylase. L-ornithine is combined with carbamoyl-phosphate, formed by by glutamine-dependent carbamoyl phosphate synthetase (CPS), to form L-citrulline. Ultimately, argininosuccinate synthase (ASSY) catalyzes the interconversion of L-citrulline and L-aspartate to L-arginino-succinate. Release of fumarate by argininosuccinate lyase (ARLY) yields L-arginine. Enzyme commission (EC) numbers are provided **(A)**. Expression levels represented in the metabolic map were calculated as the log_2_[FC] relative to those of the housekeeper EF-1α; values for *D. glomerata* appear in normal font over long arrows; for *C. thyrsiflorus* values are given in bold italic over short arrows. A metabolic heatmap is provided. Contig mean TPM values are plotted in **(B)**. **(C)** Displays the transcript abundance quantified by RT-qPCR for NAOAT and CPS. Gene expression levels in roots (R) and nodules (N) are relative to those of EF-1α. The median and IQR of three biological replicates is shown. Species name is given. Genes nomenclature used is based on Winter et al. (2015).

Figure S6 Biosynthesis of thiamine. Thiamine (vitamin B1) is assembled by the condensation of pyrimidine and thiazole moieties. In *Arabidopsis thaliana*, THIC and TH1 catalyze the biosynthesis of the pyrimidine moiety, whereas thiazole is synthesized by the bifunctional THI1 enzyme (designated THI4 in other organisms; Godoi et al., 2006). After the coupling of the two precursors by TH1, thiamine phosphate is dephosphorylated to thiamine, which is subsequently dephosphorylated by TPK1 to its active form, thiamine diphosphate. Expression levels given in the metabolic map were calculated as the log_2_[FC] relative to those of EF-1α estimated from the mean TPM of five (*D. glomerata*, long arrows with values written in normal font) and three (*C. thyrsiflorus*, short arrows with values written in bold italic) independent RNA preparations. EC numbers and Arabidopsis thaliana homologs are given. Explanatory heatmap is provided.

Figure S7 Tetrahydrofolate (THF) biosynthetic pathway. Generically known as folic acid, “folate” or vitamin B9, THF is assembled by three moieties: a pterin ring, p-aminobenzoate (pABA), and a glutamate tail. Enzymes involved in the synthesis of the pterin moity are: GTP cyclohydrolase I (GTPCHI), Nudix hydrolase (NUDT1), and dihydroneopterin aldolase (DHNA). In parallel, the pABA moiety is synthesized from chorismate. Enzymes operating in this branch are amino deoxychorismate synthase (ADCS) and 4-amino deoxychorismate lyase (ADCL). The coupling of these two branches is mediated by dihydropteroate synthase (DHPS), whose product is converted by dihydrofolate synthase (DHFS), followed by dihydrofolate reductase (DHFR). Expression levels given in the metabolic map were calculated as the log_2_[FC] relative to those of EF-1α based on the mean TPM of five (D. glomerata, long arrows with values written in normal font) and three (C. thyrsiflorus, short arrows with values written in bold italics) independent RNA preparations. Explanatory heatmap is provided. EC numbers and Arabidopsis thaliana gene orthologs are given. Nomenclature is based on Gorelova et al. (2017).

Figure S8 Hierarchical screening of transcripts encoding for members of the S8 subtilisin-like peptidase family from nodules of *D. glomerata* **(A)** and *C. thyrsiflorus* **(B)**. Heatmap *omic* colors translate the TPM in each library as low (white), medium low (green), medium (dark green), medium high (violet), and high (purple). Species names are provided. After doi: 10.17045/sthlmuni.6181760.v1.

Figure S9 Maximum-likelihood phylogenetic tree of cucumisins. *Datisca glomerata* and *Ceanothus thyrsiflorus* candidates (in bold) were queried for phylogenetic placement using the comprehensive dataset of Taylor and Qiu (2017) (see section “Materials and Methods”). When possible, GenBank accessions are provided after species name. Otherwise, an asterisk (^∗^) indicates the nomenclature used by Taylor and Qiu (2017).

Figure S10 *Datisca glomerata* and *Ceanothus thyrsiflorus* nodules express putative orthologs of Nod factor receptors from legumes. Maximum-likelihood phylogenetic reconstruction of LysM-type and LysM receptor kinases is shown. Proteins encoded by genes identified based on studies of legume mutants are highlighted in color. Acronyms of included species are: At, *Arabidopsis thaliana*; Ca, *Cicer arietinum*; Cp, *Cucurbita pepo*; Fv, *Fragaria vesca*; Gm, *Glycine max*; Lj, *Lotus japonicus*; Me, *Manihot esculenta*; Mt, *Medicago truncatula*; Mn, *Morus notabilis*; Os, *Oryza sativa*; Ps, *Pisum sativum*; Pp, *Prunus persica*; Rc, *Ricinus communis*; Sl, *Solanum lycopersicum*; Sb, *Sorghum bicolor*; Tc, *Theobroma cacao*; Vr, *Vigna radiata*; and Zj, *Ziziphus jujuba*. Different orthogroups are distinguished by color: EPR3 in purple, NFR1 in green, and NFR5 in blue. Candidate orthologs from *D. glomerata* and *C. thyrsiflorus* are given in black in bold print. GenBank accessions are given.

Table S1 *Datisca glomerata* pre-assembly summary: raw reads and filtering stages evaluation across five libraries. The last row displays the representation of each library into the Trinity assembly.

Table S2 Primers used in this study for *Datisca glomerata.*

Table S3 Primers used in this study for *Ceanothus thyrsiflorus*.

Table S4 Target taxa used for phylogenetic reconstruction of LysM and LysM-type receptors kinases.

Table S5 List of genes analysed by RT-qPCR. Gene names, protein functions, number of contig in assembly, GenBank accession number and, if available, E.C. number are given.

Table S6 *p* values for RT-qPCR analysis. For all RT-qPCT analysis, *p*-values and adjusted (FDR) *p*-values are listed.
