# Supplementary figures and images for: Comparative Analysis of the Nodule Transcriptomes of Ceanothus thyrsiflorus (Rhamnaceae, Rosales) and Datisca glomerata (Datiscaceae, Cucurbitales)
Source: Front Plant Sci. 2018 Nov 14;9:1629. doi: 10.3389/fpls.2018.01629 (PMC6246699; doi:10.3389/fpls.2018.01629)

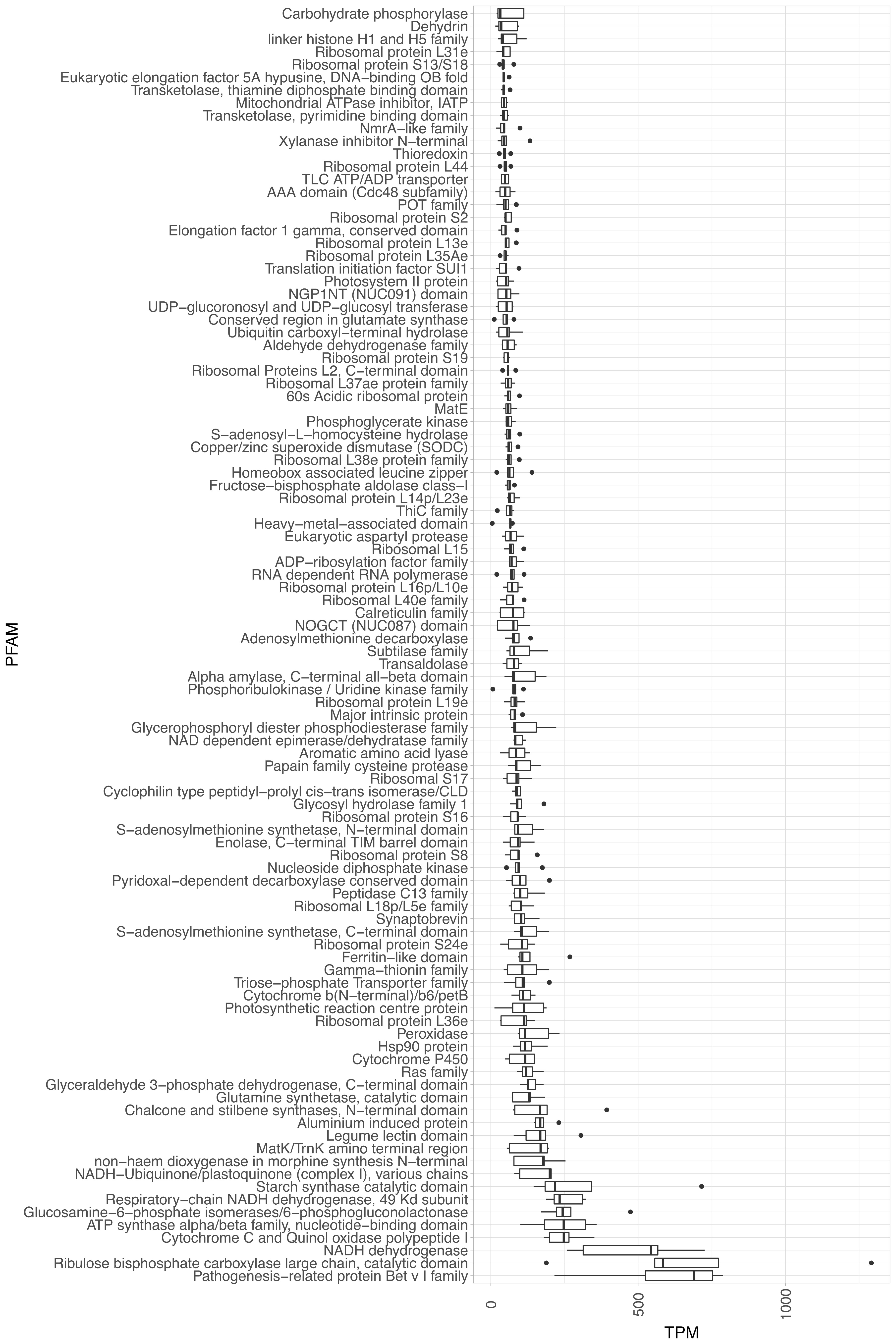

Supplement: Figure S2 — Averaged Transcript per Million reads (TPM) covering the Top 100 contigs assigned within the “biological process” category of D. glomerata. Top hits were selected based on the mean TPM of five libraries. Plotted is the median and IQR across these libraries (after doi: 10.17045/sthlmuni.6181772.v1). [file Image_2.jpeg]

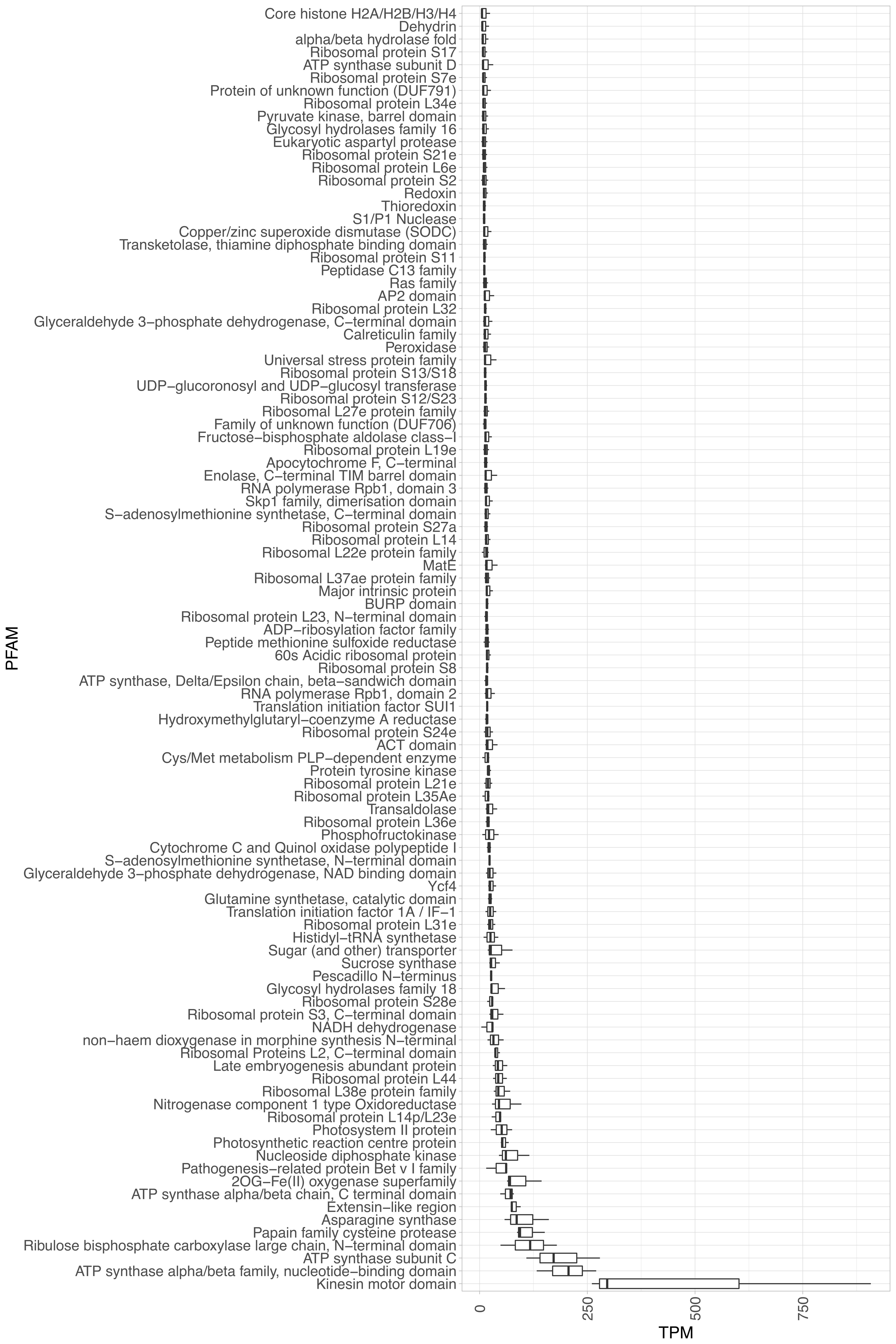

Supplement: Figure S3 — Averaged Transcript per Million reads (TPM) covering the top 100 contigs assigned within the “biological process” category of C. thyrsiflorus. Top hits were selected based on the mean TPM of three libraries. Plotted is the median and IQR across these libraries (after doi: 10.17045/sthlmuni.6181772.v1). [file Image_3.jpeg]
