# Supplementary material for: Comparative Analysis of the Nodule Transcriptomes of Ceanothus thyrsiflorus (Rhamnaceae, Rosales) and Datisca glomerata (Datiscaceae, Cucurbitales)
Source: Front Plant Sci. 2018 Nov 14;9:1629. doi: 10.3389/fpls.2018.01629 (PMC6246699; doi:10.3389/fpls.2018.01629)
Supplement: Table S1 — Datisca glomerata pre-assembly summary: raw reads and filtering stages evaluation across five libraries. The last row displays the representation of each library into the Trinity assembly. [file Table_1.docx]

## **Table S1. *Datisca glomerata* pre-assembly summary: raw reads and filtering stages evaluation across five libraries. The last row displays the representation of each library into the Trinity assembly.**

|  | Dg_201 | Dg_203 | Dg_101 | Dg_102 | Dg_103 |
| --- | --- | --- | --- | --- | --- |
| No. paired raw reads | 31,096,533 | 28,077,139 | 65,034,064 | 65,013,281 | 67,620,753 |
| % mapping *Frankia* Dg1 | 7.2 | 12.3 | 2.4 | 5.9 | 7.2 |
| No. filtered pairs | 21,661,302 | 18,806,794 | 55,893,234 | 51,631,232 | 52,802,507 |
| Reads length (nt) | 91 | 91 | 116 | 116 | 116 |
| % GC | 51 | 50 | 48 | 49 | 50 |
| % in assembly | 82.19 | 83.41 | 77.04 | 72.61 | 74.81 |
