# Supplementary material for: Comparative Analysis of the Nodule Transcriptomes of Ceanothus thyrsiflorus (Rhamnaceae, Rosales) and Datisca glomerata (Datiscaceae, Cucurbitales)
Source: Front Plant Sci. 2018 Nov 14;9:1629. doi: 10.3389/fpls.2018.01629 (PMC6246699; doi:10.3389/fpls.2018.01629)
Supplement: Table S2 — Primers used in this study for Datisca glomerata. [file Table_2.docx]

## **Table S2. Primers used in this study for *D. glomerata.***

| **gene_id** | **gene** | **Fw** | **Rv** |
| --- | --- | --- | --- |
| c47008_g1 | EF1A | ACCCTCCATTGGGTCGTTTT | GACTTTAGCCCCGGAAGGAT |
| c41833_g7 | NFR1 | CGAAGGACTACGGGCTGTTT | CGCGCTAAGATTCGTCTCCA |
| c21743_g1 | NFR5 | ATCACCAGACACGAACGCAA | TCGGGAGACTGCGCAAAATA |
| c31956_g1 | EPR3 | ACCACATCAACAATGGCCTCA | GGTAGTCTTGTCTGCTACCTACC |
| c43210_g3 | FLOT | CAACTCCGCCTCTCTCATCG | GAGGCAAACGCAGAACTAGC |
| c78522_g1 | VPY | GATGGGAACACGGCTCTTCA | ACCGTCTTCAGCATTACGCA |
| c16311_g1 | GS | GCGTCAACATTAGCGGCATC | CGAAATGCCAACGGAAGGAC |
| c36933_g1 | GOGAT | TCCCTTGGTTTAGTCGCACC | CGTTGGAGCTGATCCCTTGT |
| c11724_g1 | NIR | AACAAGGGTCACATCCTCGG | GGTTGGAGGCTTTTTCAGTCC |
| c41858_g1 | ASN1 | CTCTAGCAAAGACGGCGGAT | GTCTCAGAGCGAGTGGATCG |
| c38804_g2 | P5CS | TTCTGCTGGACATTGCCGA | CCTGTTGTGCTGCGGTAATA |
| c41827_g3 | OAT | AGGGGAAGGAGGATGTTTGC | TGGGCGCTGTATTCGTACTC |
| c31190_g1 | CPS | CCAAACCAGCAAGGAAGCAT | ATCCCAGTTATGCTGGCCAAT |
| c29417_g1 | ASSY | CACTAACTGACATGCCCCACT | TATGGCTGCGAGGTGGTTTG |
| c39485_g1 | ARLY | TCAGCTGAGTTTGCACAGAAT | AGTGTTGTGGCGTCGAGATG |
| c35449_g1 | ARGH1 | TTTGCCTGTTTTCGCCCTTC | TGCTCGAATTATGGAAGGTGGT |
| c35071_g1 | DUR3 | TTGAAGAGGACCGAGGGGAG | GAAACTTCTTCCCGCCGACT |
| c26239_g1 | NAOAT | GTCGTCGGATGCCTCAATGT | GACCAGCACTTTCCCCTCAT |
| c37525_g2 | ARGJ | CAGAAAGGCTGGTGAGACCC | TCGTAACTTAGATCGCAGCCC |
| c38233_g2 | THIC | CCCAGGTGAACTCGTCGAAA | TGTTTCCCCAAGAGCACGAA |
| c53044_g1 | THI4 | GCGCACTACCATAGCAGAGA | AGCAAGAACCCGTCTGTTCA |
| c4839_g1 | NUDT1 | TCAAGTTCATGCTCTCCCCAC | CTGAGGATGTGCCAGTCGAT |
| c69623_g1 | ADCL | GATCGGGTGTTCATCCCACA | TTACGGTGGACGAAGCCAAA |
| c29874_g1 | GLB3 | CATTTCCGGTCACGCATCAG | TGCGTCAATATCAGGGGTGC |
| c28519_g1 | HBL1 | AGCCCCTTGTTTATGGACATCT | CAACGAAGAGCCATTTCTGAGTG |
| c35069_g1 | HBL2 | GCGTGAGCTTTGAGCTTAGGAT | GAGATAGCTCCAGCTGCAAAGAA |
| c44623_g1 | SODC | CATCGGAAGGGCTGTTGTTG | CACCAGCATTCCCAGTCGT |
| c33755_g2 | SODM | GGCCCAACCTCATCATCCTAA | TAGTCATCCACAGACGGAACC |
| c86876_g1 | PER60 | TGCACCTCAAAACCTAGGCG | GCACAAGAGACAAGACCAGGA |
| c15041_g1 | PER42 | TGAAAGAGAGTGTCCTGGGG | TACGGTCCTCCAAGCGAAAC |
| c22936_g1 | APX1 | ACTTTTGTCCGGTGGGAAGG | CACCAGAGGGCGGAATACAG |
| c3666_g1 | APX2 | GGGAAAGAGCTACCCGACTG | AGCATAAGAGGAGCGCAGTT |
| c28693_g1 | DHAR2 | CCTGCTCTGACCCATCGTTAG | CCCTTCTCTCGCTACTCCTCT |
| c78212_g1 | GSTXC | TGCGTTTGTGATAATGGCGG | CTCAGCCAAAGCGATCCTCA |
| c69924_g1 | GPX4 | GTGGAATTTGCTTGCACTCGT | GTACAGTGGGGCAGCTTTCT |
| c70007_g1 | GSTUH | CACAGATCACACTACGCACCT | AGAGAGCACGCGGATCATAC |
| c61839_g1 | MDARS | GCAACCCCCTCCTTGGAAATA | GTGATAGTTGGCGGTGGTGT |
| c22224_g1 | RBS | TCTGCCTCCTCTCTCTGATGAA | GCCCCACCTCATCAAACTCA |
| c78170_g2 | RBL | GGACAACTGTGTGGACCGAT | GCAACAGGCTCGATGGAGTA |
| c88499_g1 | STEP2 | CGACAGCTTCTCTCGGACTT | CCCCTCCGTTCGATAGACCT |
| c69584_g1 | CUCM1 | GCAAGCAAGGGTACAACACC | TTAGGTCCCATCCCCTTCCG |
| c61424_g1 | Dg12 | TTCAGGGGAAATCACGAGGC | GCGGCTTCTCAACTGTTTGG |
