# Supplementary material for: Comparative Analysis of the Nodule Transcriptomes of Ceanothus thyrsiflorus (Rhamnaceae, Rosales) and Datisca glomerata (Datiscaceae, Cucurbitales)
Source: Front Plant Sci. 2018 Nov 14;9:1629. doi: 10.3389/fpls.2018.01629 (PMC6246699; doi:10.3389/fpls.2018.01629)
Supplement: Table S3 — Primers used in this study for Ceanothus thyrsiflorus. [file Table_3.docx]

**Table S3. Primers used in this study for *C. thyrsiflorus.***

| **gene_id** | **gene** | **Fw** | **Rv** |
| --- | --- | --- | --- |
| c24602_g1 | EF1A | TTCTTGGCAGCAGACTTGGT | TGACATGCGCCAGACAGTT |
| c26717_g1 | NFR1 | ACAATCGAGGCGTTGACACA | TCGCTATTGCCACACGAACA |
| c37549_g1 | NFR5 | CAGTGGGGCTTCCATTGTTG | AATCCGCCGGTCTTTCCATT |
| c39118_g2 | EPR3 | TTCGCGGAGCTAAAGGTGTT | CATCTCCACAGGCATACCCC |
| c19475_g1 | FLOT | GGCCGACGGAGAGTTCTATG | CCGAGAGCATCCAAAAGGGT |
| c27874_g1 | VPY | AGAAGCGAACCAGCATGGAA | GGACAAGATCAGGACGGCTT |
| c38065_g1 | LIN | CACCGGCCAGATTTTTGGTG | CGTGTTTCCCCTTTTCAGCC |
| c30458_g1 | GS | GGGCGGCTCGTTACATTTTG | CACCCTGGATTGGCTTTGGA |
| c38184_g1 | GOGAT | GCGTGCTGATAGAATTGGCG | CCATAAGGTTGACTCGCCGT |
| c20649_g1 | NIR | GGCAATCCCTCTTGATGCCT | TTTTGCCTGTTTCCTCGGGT |
| c32191_g1 | ASN1 | CGTGGACATGCTTGATGGGA | CAATAGCATCACGGGCAACG |
| c8699_g1 | P5CS | CGTCATCGTCTTTTTCGCCG | AGACCGATCCATGTCCTCCA |
| c36839_g1 | OAT | AAGCATTGGGTGGTGGAGTAA | GGTACTTCCATGCTCTCCAGG |
| c35880_g1 | CPS | CTCGCAGATTAAGGCAAGATGG | CCAACAATGTCCCATGAACGAG |
| c37782_g1 | ASSY | TTCTGGCCTATAGTGGTGGC | GCCAACATCAGCAGTAAAGCAA |
| c31877_g1 | ARLY | TGATGCAGTGTCAGACCGAG | TCTGAAGCCCACAAAACCCA |
| c31424_g1 | ARGH1 | ATGGAGGGTGGTTATGCTCG | TCTTTTGCCTTGTTCACGCC |
| c37501_g1 | DUR3 | TCAGGTGGGCTTGTCTTTGG | TGAAGGTCGTGCAGCTATGG |
| c37737_g2 | NAOAT | GAGTTCTGGGATTGCGGTGA | TTGCTGACATGGGTAAGCGT |
| c36428_g1 | ARGJ | ATCACAGCCCCATGCTTGAC | AAGAAGGCTGGTGAGACCCA |
| c24096_g1 | THIC | CATGGTTACGGGAGTGCTGA | TCCCCGCTGACAGTTTTCTT |
| c34246_g1 | THI4 | TTCTATCCGTCTCCAGCCCA | GAGGGAGTCAAAGCGGACAT |
| c39174_g2 | NUDT1 | CCTTGGGACAATCTTCCGTGT | TCAGCCTCAGCGATTACCAC |
| c35711_g2 | ADCL | GCAGGAATGGATCGCTGAGA | CGACAGCATAAAGAGCCGGT |
| c4055_g1 | GLB3 | TCTGGTGTGCTGTCCAATGC | GTCACCGTCCATTTCCCGTT |
| c17120_g1 | HBP2 | GAAGAATGCTGGAGAGCTTGC | GCTCAGGAGGAACATCAGAGT |
| c33510_g1 | SODC | TGCTAAGCTCATGTCCACCC | ACAAGCAGATTCCTCTCACCG |
| c19409_g1 | SODM | TCCCACTAGGGTTTTCCTGC | CTGGTTTCGTCACCGACTCA |
| c19257_g1 | PER42 | CATCCCAAAGCTCCTGTCCA | CCATGACTGTGCTGTCGAGT |
| c29022_g1 | CATA | CGATGACACTGGGAATTGCG | GGCAATCAAAGGGTTGGCTG |
| c4825_g1 | APX1 | ACCATTAAGCATCCCGCTGA | GAACTGCTCCTTGATGGGCT |
| c23716_g1 | DHAR1 | CTGATGGGTCAGAACAGGCTT | AGTGACCTTCTCCCCAGCAA |
| c33792_g1 | MDAR | GCAGCTAGGGAGTTTGCCA | TCATAAGGAGCCACCGCTTC |
| c11945_g1 | GPX4 | CTCCACAACCTTCCCCTCTTT | CAGCTCCCCTTTACAAGTTCCT |
| c24098_g1 | GPX6 | GGTGCATAGCGGTCAACAAC | GGTGGACTCTTTGGGGACAG |
| c25417_g1 | GPX8 | GGTCAACGACTTTCCCGTCT | AAGTCTGGCAAATGGGGCAT |
| c31503_g1 | RBS | CAACCACACCGCACAGTTTT | ACATAGCCGGACCCAGAAAC |
| c78883_g1 | RBL | GCCAGAGAATTGGGAGTTCCT | CTCGGCAATAATGAGCCAAGC |
| c35348_g1 | Ct12 | TGAAGGTGTCATTGTCGGGG | TTGGATGGCATACCGTGGTC |
| c29196_g1 | CUCM1a | GAAAGCAATCACCAGCGACC | TCCCCAAAGCGAAAGATGGG |
| c34419_g1 | CUCM1b | TTGTTGCCCCATTTTCAGCC | CCAAGCCGCAAGGATTTCAG |
| c33106_g2 | AIR3 | GGGGTCATTTCACGCTGCTA | ATCCACGGGGCAAGATTTGT |
| c31672_g1 | SUBL | ACGTCGCCAATGCAAGTCTA | GCAAACCTTGTAAGCGGCAA |
| c91758_g1 | DEF1 | GCCTTTTGCACTCAGATGACG | ACAGCGATAAAGACGGCAGG |
| c90907_g1 | DEF2 | GCTCCATAAGTTGCATACTCCCA | TGGTATGGGATTTGTTCGTGGT |
