# Supplementary material for: Comparative Analysis of the Nodule Transcriptomes of Ceanothus thyrsiflorus (Rhamnaceae, Rosales) and Datisca glomerata (Datiscaceae, Cucurbitales)
Source: Front Plant Sci. 2018 Nov 14;9:1629. doi: 10.3389/fpls.2018.01629 (PMC6246699; doi:10.3389/fpls.2018.01629)
Supplement: Table S4 — Target taxa used for phylogenetic reconstruction of LysM and LysM-type receptors kinases. [file Table_4.docx]

## **Table S4.** Target taxa used for phylogenetic reconstruction of LysM and LysM-type receptors kinases.

| **Species name** | **Taxon ID** |
| --- | --- |
| *Arabidopsis thaliana* | 3702 |
| *Cicer arietinum* | 3827 |
| *Cucurbita pepo subsp. pepo* | 3664 |
| *Fragaria vesca subsp. Vesca* | 101020 |
| *Glycine max* | 3847 |
| *Lotus japonicus* | 34305 |
| *Manihot esculenta* | 3983 |
| *Medicago truncatula* | 3880 |
| *Morus notabilis* | 981085 |
| *Oryza sativa Japonica group* | 39947 |
| *Prunus persica* | 3760 |
| *Ricinus communis* | 3988 |
| *Solanum lycopersicum* | 4081 |
| *Sorghum bicolor subsp. bicolor* | 4558 |
| *Theobroma cacao* | 3641 |
| *Vigna radiata var. radiate* | 3916 |
| *Ziziphus jujuba* | 326968 |
